# Changes of Crocin and Other Crocetin Glycosides in Saffron Through Cooking Models, and Discovery of Rare Crocetin Glycosides in the Yellow Flowers of *Freesia Hybrida*

**DOI:** 10.3389/fnut.2022.885412

**Published:** 2022-07-14

**Authors:** Kazutoshi Shindo, Yuka Sakemi, Saki Shimode, Chiharu Takagi, Yohei Uwagaki, Jun-ichiro Hattan, Miu Akao, Shiori Usui, Ayako Kiyokawa, Masako Komaki, Minoru Murahama, Miho Takemura, Isamu Ishikawa, Norihiko Misawa

**Affiliations:** ^1^Department of Food and Nutrition, Japan Women's University, Tokyo, Japan; ^2^CaroProTech Corporation, Nomi-shi, Japan; ^3^Research Institute for Bioresources and Biotechnology, Ishikawa Prefectural University, Nonoichi-shi, Japan; ^4^Ishikawa Agriculture and Forestry Research Center, Kanazawa, Japan

**Keywords:** saffron, freesia, crocin, crocetin neapolitanosyl ester, cooking

## Abstract

Crocetin glycosides such as crocin are noted as functional food materials since the preventive effects of crocin have been reported against chronic disease and cancer. However, it is unclear how these apocarotenoids are structurally changed through cooking for our intake. We examined such changes in crocetin glycosides (crocin, tricrocin, and crocin-3) contained in saffron (stigmas of *Crocus sativus*) through cooking models. These glycosides were almost kept stable in boiling for 20 min (a boiled cooking model), while hydrolysis of the ester linkage between glucose and the crocetin aglycone occurred in a grilled cooking model (180°C, 5 min), along with a 13-*cis* isomerization reaction in a part of crocetin subsequently generated. We further here revealed that the yellow petals of freesia (*Freesia* x *hybrida*) with yellow flowers accumulate two unique crocetin glycosides, which were identified to be crocetin (mono)neapolitanosyl ester and crocetin dineapolitanosyl ester. A similar result as above was obtained on their changes through the cooking models. Utility applications of the freesia flowers as edible flowers are also suggested in this study. Additionally, we evaluated singlet oxygen (^1^O_2_)-quenching activities of the crocetin glycosides contained in saffron and freesia, and crocetin and 13-*cis* crocetin contained in the grilled saffron, indicating that they possessed moderate ^1^O_2_-quenching activities (IC_50_ 24–64 μM).

**Graphical Abstract:**
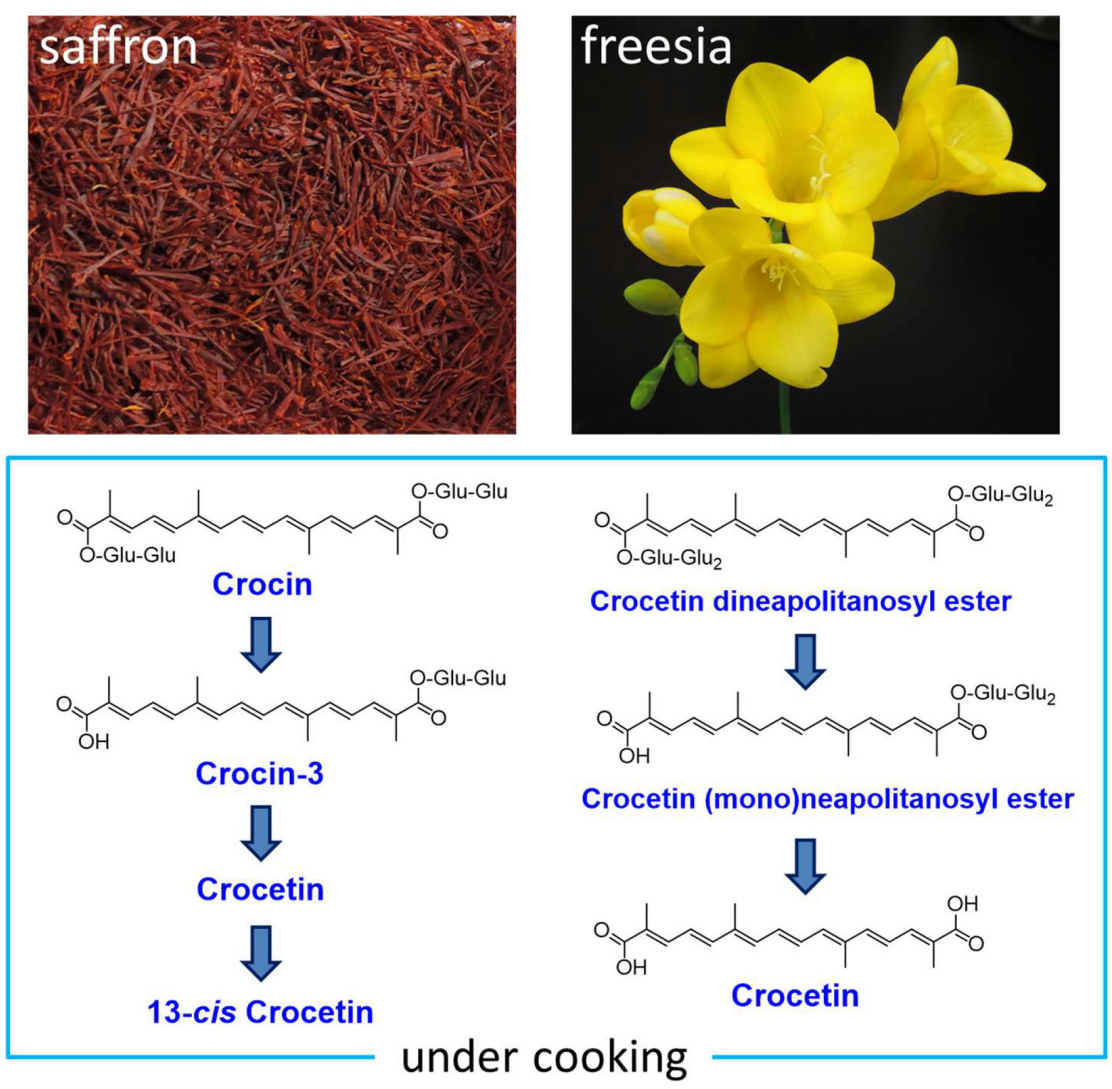


## Introduction

**Figure 1:**
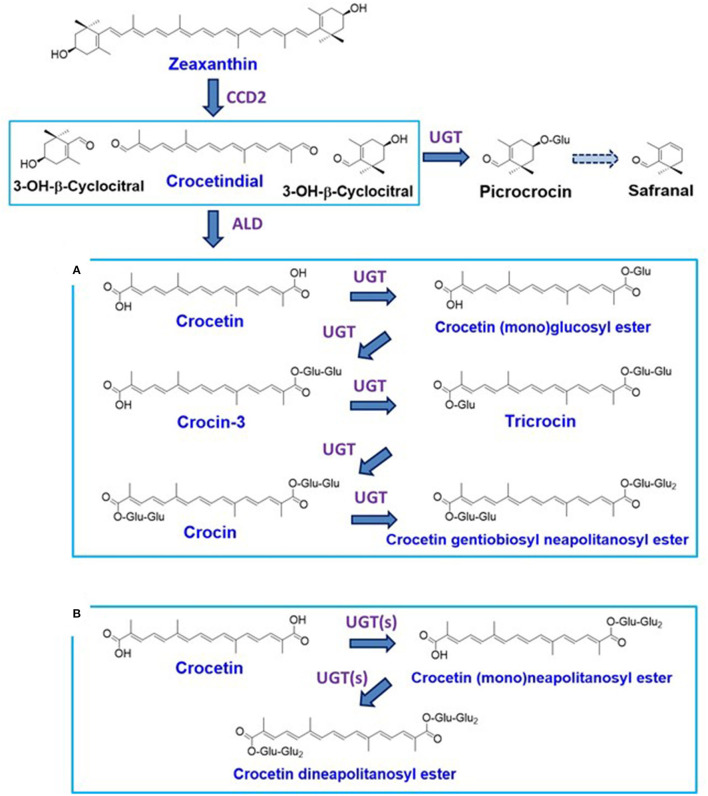
Carotenoid metabolism in saffron [stigmas of *Crocus sativus*; **(A)**] and the yellow flowers of freesia [*Freesia* x *hybrida*; **(B)**].

Crocetin is an apocarotenoid composed of 20 carbons, and its digentiobiosyl ester is called crocin, which is the major pigment contained in the dried red stigmas of saffron plants (*Crocus sativus* L.) and gardenia fruits (*Gardenia jasminoides* Ellis), which belong to the Iridaceae and Rubiaceae families, respectively (1). Along with crocin, these plant organs have been reported to accompany several minor components of crocetin glycosides, i.e., crocetin (mono)glucosyl ester, crocin-3 [crocetin (mono)gentiobiosyl ester], tricrocin (crocetin gentiobiosyl glucosyl ester), and crocetin gentiobiosyl neapolitanosyl ester (1, 2). The biosynthetic pathways of these apocarotenoids are shown in Figure 1A (3, 4). Saffron is an expensive spice, which has widely been used in cooking as a water-soluble yellow colorant with a distinctive flavor (5). Crocin and relevant glycosides are noted as functional food materials since the preventive effects of crocin have been reported against chronic diseases such as diabetes, fatty liver, and autoimmune diseases, and cancer such as colon cancer (6–9). It is however unclear how crocin and the relevant glycosides are structurally changed through cooking (boiling and grilling) for our intake, although changes of carotenoids that are contained in maize and vegetables through cooking were examined in a few reports (10, 11). In this study, such a change is examined on saffron. In addition, we evaluate singlet oxygen (^1^O_2_)-quenching activities of the generated compounds.

**Figure 2:**
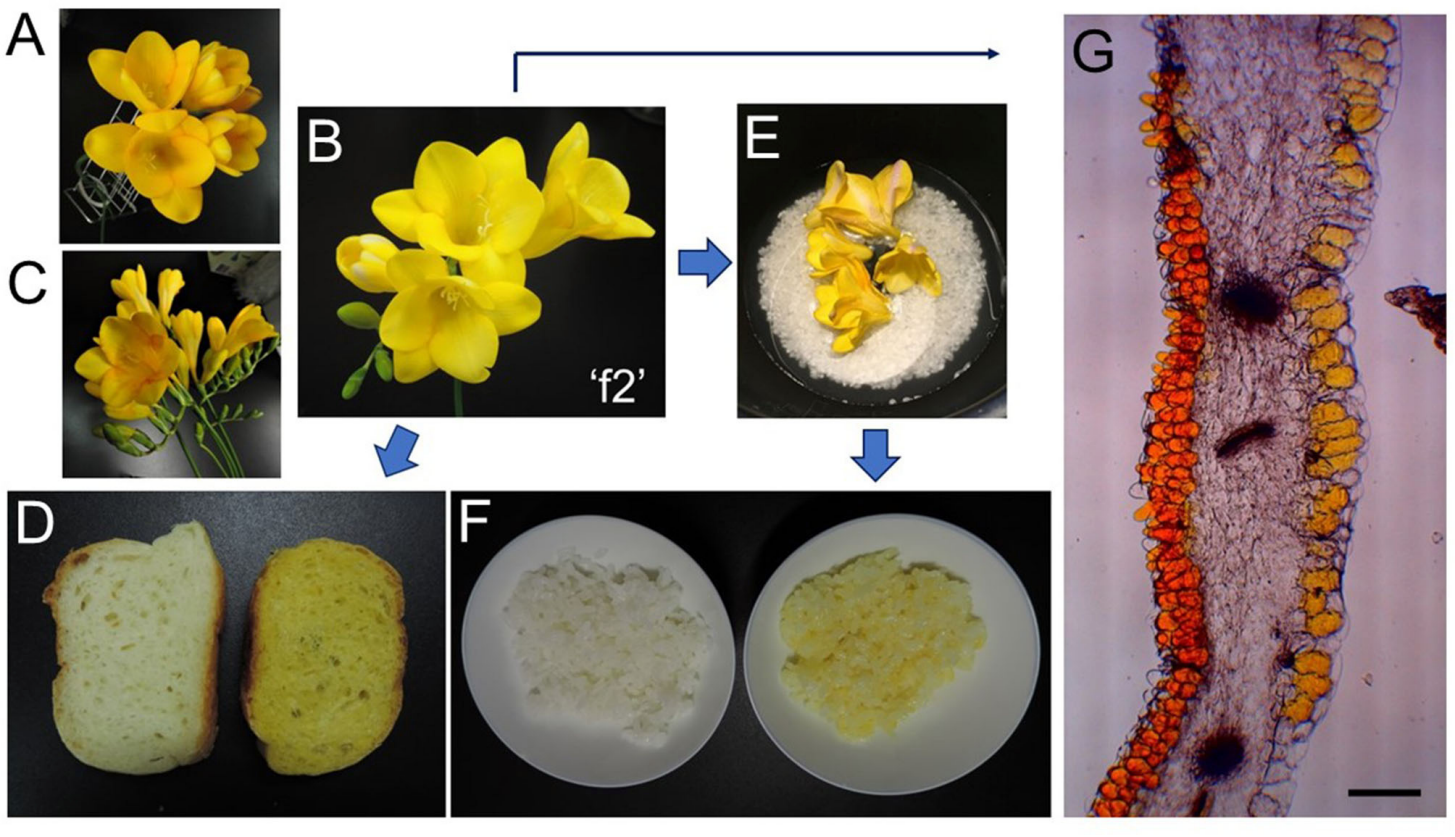
Freesia-relevant pictures: **(A)**, freesia (*Freesia* x *hybrida* L.) cultivar “Aladin;” **(B)**, freesia cultivar “Ishikawa f2 go” (“f2”); **(C)**, freesia cultivar “Kayak;” **(D)**, bread baked without (left) and with (right) freesia “f2” flowers; **(E)**, before cooking with freesia “f2” flowers by a rice cooker; **(F)**, rice cooked without (left) and with (right) freesia “f2” flowers; **(G)**, microscopy image of a freesia “f2” petal (Bar represents 200 μm).

Freesia (*Freesia* x *hybrida*; the Iridaceae family) is a popular flowering plant of South African (the Cape Province area) origin as the cut flowers (12). Many cultivars of freesia plants, whose flowers retain a variety of colors, shapes, and fragrances, have been generated up to the present (13). In Japan, freesia cultivars with yellow flowers maintain an 80% share of the market. Among them, “Aladin” (Figure 2A), which retains big yellow flowers, has been the most cultivated freesia cultivar since the 1990s. Cultivar “Ishikawa f2 go” (“f2”; Figure 2B) was established by crossing cultivars, “Aladin” and “Rapid Yellow” that retains a characteristic suitable for early forcing, and by selecting one seedling among consequently generated 222 seedlings (14). This study reveals that yellow pigments in the yellow petals of freesia [cultivars “f2” and “Aladin” in addition to cultivar “Kayak” (Figure 2C)] are unique apocarotenoids, crocetin (mono)neapolitanosyl ester and crocetin dineapolitanosyl ester (Figure 1B). Plants that produce such apocarotenoids have not been reported so far. Moreover, this is the first report on plants that include the (mono)neapolitanosyl ester. According to Edible Flowers Guide by Thompson & Morgan (https://www.thompson-morgan.com/edible-flowers), freesia is positioned as an unusual edible flower, and described to be “Infused in a tisane with lemon juice and zest. The peppery scent and bold color are a perfect pick-me-up.” Utility applications of the freesia flowers as edible flowers are also suggested in this study.

## Materials and Methods

### Plant Materials

Dried red pistils containing stigmas (called “saffron”) from saffron plants (*Crocus sativus* L.) that had been cultivated in Spain were purchased at a spice-special store in Tokyo, Japan. As for freesia (*Freesia* x *hybrida*), we used cultivars “Ishikawa f2 go” (“f2”), “Kayak,” and “Aradin.” Cultivars “f2” and “Kayak” were grown in a greenhouse, and their flowers were harvested in middle and late March, respectively. Cultivar “Aladin” was purchased at a floral shop in Ibaragi, Japan.

Microscopy pictures of the “f2” petal were recorded by BH2 (Olympus) after cutting it in 3% agar with MicroSlicer ZERO-1 (Dosaka EM, Kyoto, Japan).

### Reagents

Dichloromethane (CH_2_Cl_2_), methanol (MeOH), acetonitrile (CH_3_CN), ethyl acetate (EtOAc), and chloroform of analytical grade were purchased from Wako Pure Chemicals Industries, Ltd. (Osaka, Japan) or Kanto Chemical Co. Inc. (Tokyo, Japan). The other chemicals were purchased from Sigma-Aldrich (St. Louis, MO, United States).

### GC-MS Analysis

In order to detect volatile components of saffron and freesia “f2” flowers, we carried out a gas chromatography-mass spectrometry (GC-MS) analysis. Each 0.1 g of their dried materials was ground with an addition of 0.3 ml of water (H_2_O) and 0.3 ml of MeOH, subsequently extracted with 0.6 ml of chloroform (by mixing and centrifugation), and the chloroform extraction was repeated by adding 0.5 ml of H_2_O, and finally, each chloroform phase was concentrated to threefold by decompression. The prepared samples were subjected to GC-MS using Shimadzu GCMS-QP5050 (Shimadzu, Kyoto, Japan), as described (15).

### NMR and MS Analysis

Nuclear magnetic resonance (NMR) spectra (^1^H and ^13^C NMR, ^1^H-^1^H DQF COSY, HMQC, HMBC, and NOESY) were measured using an AVANCE 400 MHz NMR spectrometer (Bruker, Rheinstettern, Germany) utilizing standard programs in TopSpin1.3. Chemical shifts were referenced to solvent signals. HR-ESI-MS (+) was measured using a JMS-T100LP mass spectrometer (JEOL, Tokyo, Japan) and accumulated mass calibration was performed using reserpine [C_33_H_41_N_2_O_9_, *m/z* 609.2812044 (M+H)^+^].

### Cooking Models and Actual Cooking

As a boiled cooking model, saffron (dried; 0.08 g) or freesia petals [1.8 g (fresh weight)] were added to boiled water (50 ml) and boiled for 20 min. As a grilled cooking model, saffron (dried; 0.08 g) or freesia petals [1.8 g (fresh weight)] were added to olive oil (7.5 ml) and grilled at 180°C for 5 min.

A paella-type cooking was adopted as an example of the boiled dishes with saffron, as follows: Saffron (0.08 g)-including water (300 ml), polished rice (500 g), and olive oil (30 ml) were put together on a frying pan, and the materials were boiled under medium heat for 20 min. A pilaf-type cooking was adopted as an example of the grilled dishes with saffron, as follows: Olive oil (30 ml), polished rice (500 g), and saffron (0.08 g) were grilled on a frying pan under medium heat for 10 min. Then, water (300 ml) was added to the frying pan and the materials were boiled for 15 min.

Rice was cooked in a rice cooker, using polished rice (Japonica; 75 g), water (90 ml), and “f2” flowers (5 g fresh weight) (Figure 2E). After cooking, the flower debris was eliminated (Figure 2F right). Bread was baked as follows: Wheat powder (100 g), water (60 ml), NaCl (1.5 g), budding yeast (powder; 1 g), and dried powder from “f2” flowers (10 g fresh weight) by microwave were mixed and left for 2 h 20 min. The material was incubated for 1 h at 37°C, and baked for 21 min at 220°C (Figure 2D right).

### Extraction of Apocarotenoids From Cooked Materials and Their HPLC-DAD Analysis

Each sample obtained by cooking had an addition of 100 μL of 9,10-dimethylanthracene solution (10 mg/1 ml CH_2_Cl_2_) as an internal standard to confirm the recovery rate of apocarotenoids. Each of the cooked materials as the cooking models was extracted by CH_2_Cl_2_-MeOH (1:1) (V:V) (20 ml), MeOH (20 ml), and 50% (V/V) MeOH (20 ml) in a stepwise manner by stirring for 30 min at 20°C. All the extracts (total 60 ml) were combined and concentrated to dryness *in vacuo*.

Each of the actually cooked materials [the paella (boiled) and pilaf (grilled) types] was freeze-dried and powdered by a mill. The powder was extracted by CH_2_Cl_2_-MeOH (1:1) (1 L), MeOH (1 L), and 50% (V/V) MeOH (1 L) in a stepwise manner by stirring for 30 min at 20°C, and all the extracts (total 3 L) were combined and concentrated to dryness *in vacuo*.

Each extract was dissolved into MeOH (10 ml), and 2.5 μL (saffron) or 20μL (freesia) of the supernatant was analyzed by high-performance liquid chromatography-diode array detector (HPLC-DAD) [column, PEGASIL ODS SP100 (4.6 × 150 mm, Senshu Scientific Co. Ltd., Tokyo, Japan); solvents: A 5% (V/V) CH_3_CN containing 20 mM phospholic acid, B 95% (V/V) CH_3_CN containing 20 mM phospholic acid. 0 → 5 min 100% A, 5 → 18 min 100% A → 100% B linear gradient, 18–30 min 100% B, flow rate, 3.0 ml/min; detection, DAD; monitored at 250–600 nm].

### Purification of Individual Apocarotenoids (1, 2, 3, and 4) From Saffron

Saffron (0.22 g) was extracted for 30 min at 20°C by stirring with CH_2_Cl_2_-MeOH (1:1) (V:V) (100 ml), MeOH (100 ml), and 50% (V/V) MeOH (100 ml) in a stepwise manner. CH_2_Cl_2_-MeOH (1:1) extract was concentrated to dryness to give a yellow oil, and the yellow oil was applied on a diol column chromatography (10 × 150 mm, CHROMATOREX DIOL MB 100-40/75, Fuji Silysia Chemical Ltd., Aichi, Japan) with *n*-hexane-CH_2_Cl_2_ (5:1) (V:V) and developed with the same solvent. The elution fractions containing yellow pigment were combined and concentrated to dryness (4.7 mg), and further purified by ODS preparative HPLC [column, Develosil C30-UG-5 (10 × 250 mm, Nomura Chemical Co. Ltd., Aichi, Japan); solvent, CH_2_Cl_2_-CH_3_CN (95:5) (V:V); flow rate, 3.0 ml/min; detection, DAD; monitored at 250–600 nm]. The peak eluted at 9.5 min was collected to afford pure **4** (0.4 mg).

MeOH and 50% MeOH extracts were combined and concentrated to dryness (38.2 mg), and **1**–**3** in the extract were purified by ODS preparative HPLC [column, ADME ODS (10 × 250 mm, Osaka Soda Co. Ltd., Osaka, Japan); solvent, 50% (V/V) CH_3_CN containing 0.1% (V/V) trifluoro acetic acid (TFA); flow rate, 3.0 ml/min; detection, DAD; monitored at 250–600 nm]. In this HPLC, the mixture of **1** and **2** was eluted at 3.0 min, and **3** was eluted at 5.0 min. The peak of **3** eluted at 5.0 min was collected and concentrated in a small volume to remove CH_3_CN and finally freeze-dried to afford pure **3** (7.1 mg). The peak eluted at 3.0 min was also collected and freeze-dried to give yellow oil containing **1** and **2**. This yellow oil containing **1** and **2** was further purified by ODS preparative HPLC [column, PEGASIL ODS SP100 (20 × 250 mm); solvent, 30% (V/V) CH_3_CN; flow rate, 8.0 ml/min; detection, DAD; monitored at 250–600 nm]. In this HPLC, **1** and **2** were eluted at 8.0 min (12.8 mg) and 13.0 min (11.4 mg), respectively, as pure compounds.

### Purification of Individual Apocarotenoids (5 and 6) From Grilled Saffron

Saffron (0.22 g) in olive oil (10 ml) was grilled for 1 min in a frying pan with medium heat and extracted with CH_2_Cl_2_-MeOH (1:1) (V:V) (100 ml) twice. The extract (200 ml) was concentrated to dryness, added *n*-hexane (30 ml), and mixed well. The precipitate containing apocarotenoids was purified by ODS preparative ODS HPLC [column, Develosil C30-UG-5 (20 × 250 mm); solvent, 85% (V/V) CH_3_CN containing 0.1% (V/V) TFA; flow rate, 8.0 ml/min; detection, DAD; monitored at 250-600 nm]. In this HPLC, **5** and **6** were eluted at 12.0 min (3.2 mg) and 17.0 min (2.1 mg), respectively, as pure compounds.

### Purification of Yellow Pigments From Freesia Flowers

Freesia (*Freesia* x *hybrida*) “f2” flowers (179.3 g) were freeze-dried and powdered by mill (18.6 g). This powder was extracted for 30 min by stirring with CH_2_Cl_2_-MeOH (1:1) (V:V) (1 L), MeOH (1 L), 80% (V/V) MeOH (1 L), and 50% (V/V) MeOH (1 L) in a stepwise manner.

The CH_2_Cl_2_-MeOH (1:1) and MeOH extracts were combined and concentrated to dryness *in vacuo* (14.50 g). This extract was dissolved in H_2_O (300 ml) and partitioned with EtOAc (300 ml) twice, and a water layer containing yellow pigment was collected. The water layer was concentrated to 200 ml to remove EtOAc, 1 M HCl (10 ml) was added to adjust at pH3, and adsorbed to HP-20 column chromatography (50 × 150 mm, Mitsubishi Chemical Corporation, Tokyo, Japan). The HP-20 column was washed with H_2_O (750 ml) and 50% (V/V) MeOH (750 ml), and the yellow compound was eluted with MeOH (750 ml). The MeOH eluate was concentrated to dryness to obtain yellow oil (1.11 g). The yellow oil was mixed with 15 ml of CH_2_Cl_2_, and the insoluble yellow sediment was collected (repeated twice). The obtained red sediment was dried (0.91 g), dissolved in 3 ml of 50% MeOH, and further purified using preparative ODS HPLC (column, Develosil C30-UG-5 (10 × 250 mm); solvent, 30% (V/V) CH_3_CN + 0.1% (V/V) TFA; flow rate, 3.0 ml/min; detection, DAD; monitored at 200–600 nm). The yellow compound was eluted at 17.8 min as pure compound (**7**) (27.1 mg).

The 80% MeOH (including compound **7** also) and 50% MeOH extracts were combined and concentrated in a small volume (300 ml) to remove MeOH and partitioned with EtOAc (300 ml) twice, and the water layer containing yellow pigment was collected. The water layer was concentrated to 200 ml to remove EtOAc, and adsorbed to HP-20 column chromatography (50 × 150 mm). The HP-20 column was washed with H_2_O (750 ml) and the yellow compound was eluted with 50% (V/V) MeOH (750 ml). The 50% MeOH eluate was concentrated to dryness to obtain yellow oil (0.75 g). The yellow oil was then purified using preparative ODS HPLC [column, Develosil C30-UG-5 (10 × 250 mm); solvent, 20% (V/V) CH_3_CN + 0.1% (V/V) TFA; flow rate, 3.0 ml/min; detection, DAD; monitored at 200–600 nm]. The yellow compound was eluted at 15.3 min as pure compound (**8**) (14.0 mg).

### Physico-Chemical Properties of Crocetin Neapolitanosyl Ester (7)

HR-ESI-MS (+) *m/z* 837.31834 (C_38_H_54_O_19_Na calcd. for 837.31570, Δ 3.16 ppm). UV-Vis λmax (ε) in MeOH 254 (9200), 315 (6800), 407 (43000), 428 (62000), 452 (56000). ^1^H NMR (CD_3_OD) δ: 1.96 (3H, s, H-19), 2.00 (9H, s, H-20, H-19', and H-20'), 3.15 (1H, m, H-5”'), 3.17 (1H, m, H-2”'), 3.21 (1H, m, H-2””), 3.24 (2H, H-4”” and H-5””), 3.36 (2H, H-3”' and H-3””), 3.37 (1H, m, H-4”'), 3.54 (1H, m, H-4”), 3.57 (1H, m, H-5”), 3.65 (2H, H-6”'b and H-6””b), 3.73 (1H, m, H-2”), 3.78 (1H, m, H-3”), 3.80 (1H, m, H-6”b), 3.83 (2H, H-6”'a and H-6””a), 4.15 (1H, m, H-6”a), 4.32 (1H, d, *J* = 7.6 Hz, H-1””), 4.59 (1H, d, *J* = 7.8 Hz, H-1”'), 5.65 (1H, d, *J* = 7.8 Hz, H-1”), 6.47 (3H, H-14, H-9', and H-14'), 6.62 (1H, m, H-11'), 6.64 (1H, m, H-11), 6.70 (1H, m, H-8'), 6.78 (1H, m, H-12), 6.80 (2H, H-15 and H-15'), 7.29 (1H, d, *J* = 9.1 Hz, H-10'), 7.42 (1H, d, *J* = 11.9 Hz, H-10). ^13^C NMR (CD_3_OD) δ: 12.7 (C-19), 12.7 (C-19'), 12.9 (C-20), 12.9 (C-20'), 62.2 (C-6”'), 62.6 (C-6””), 69.4 (C-6”), 70.6 (C-4”), 71.0 (C-4”'), 71.5 (C-4””), 75.0 (C-2””), 76.0 (C-2”'), 77.6 (C-3”), 77.6 (C-3””), 77.7 (C-5”), 77.9 (C-5”'), 77.9 (C-5””), 78.0 (C-3”'), 82.4 (C-2”), 94.6 (C-1”), 104.5 (C-1””), 105.6 (C-1”'), 124.6 (C-11), 125.0 (C-11'), 126.3 (C-9'), 127.5 (C-14'), 127.7 (C-9), 132.6 (C-12'), 133.1 (C-12), 136.7 (C-14), 137.9 (C-13), 138.2 (C-13'), 140.4 (C-10'), 142.0 (C-10), 145.0 (C-15'), 146.4 (C-15), 168.1 (C-8). 171.9 (C-8').

### ^1^O_2_-Quenching Experiment

Methylene Blue (80 μL, 25 μM in ethanol) and linoleic acid (100 μL, 0.24 M in ethanol) were added to 5 ml glass test tubes with and without 40 μl of an apocarotenoid (final concentration, 1–100 μM in ethanol). The tubes were mixed well and then illuminated at 7,000 lux and 22°C for 3 h in a Styrofoam box, after which 50 μl of the reaction mixture was removed and diluted to 1.5 ml with ethanol. OD_235_ was then measured to estimate the levels of conjugated dienes formed in the reaction (16). OD_235_ in the absence of the carotenoid was also measured as a negative control (no ^1^O_2_-quenching activity), and the ^1^O_2_ quenching activity of each apocarotenoid was then calculated relative to this reference value. The activity was determined as the IC_50_ value, representing the concentration at which 50% inhibition was observed.

## Results

### GC-MS Analysis of Volatile Components of Saffron and Freesia

**Figure 3:**
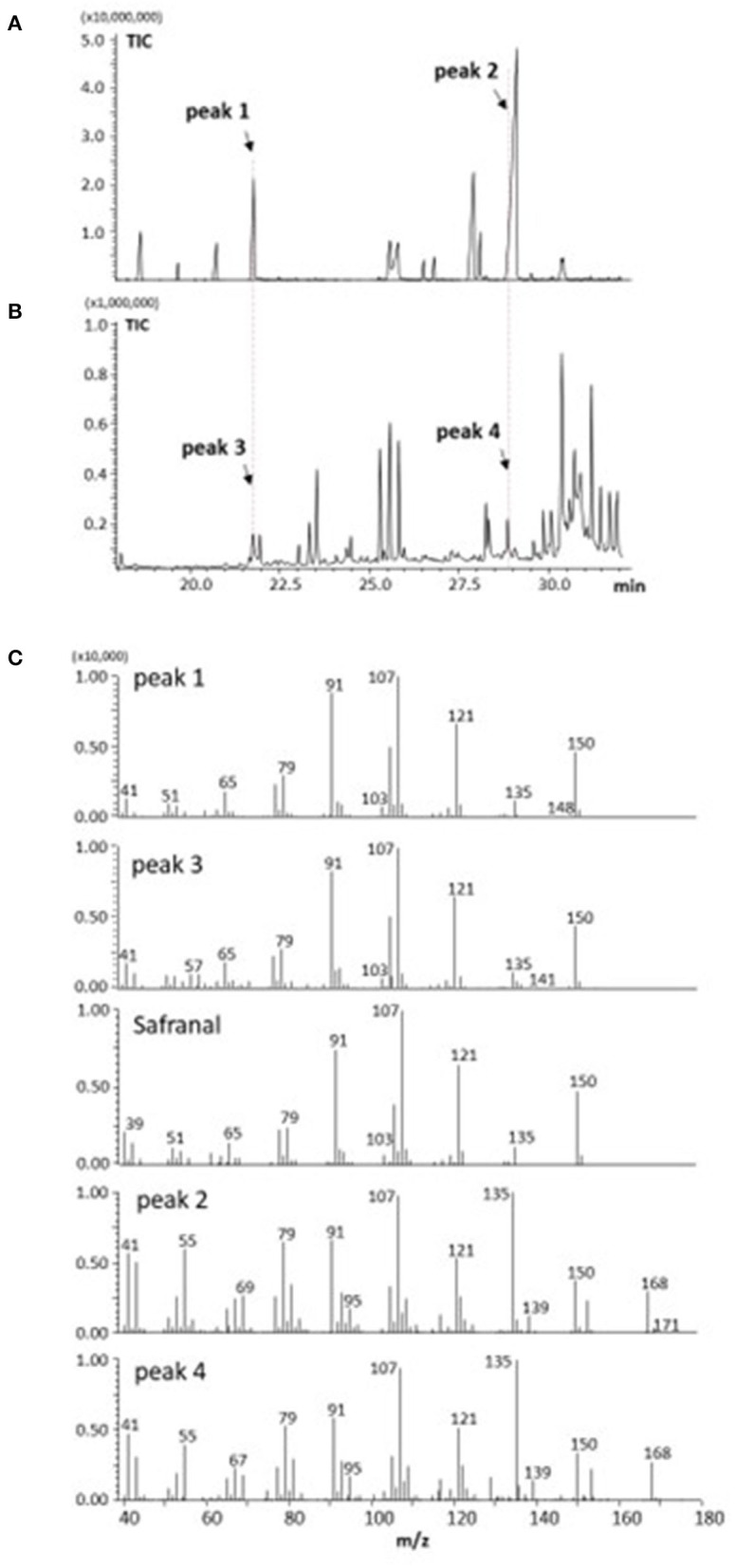
GC-MS analysis of volatile components from saffron **(A,C)** and freesia “f2” yellow-flowers **(B,C)**.

Figures 3A,B show the results of GC analysis of the MeOH-chloroform extracts of saffron (**A**) and freesia “f2” flowers (**B**). Peak 1, one of the main peaks in saffron, was found to exist in freesia as peak 3. MS spectra of peak 1 and peak 3 had the highest similarity to safranal in the database of the GC-MS system (Figure 3C). Peak 4 appearing in freesia, which corresponded to peak 2 in saffron, was considered 3-hydroxy-β-cyclocitral (C_10_H_16_O_2_; MW 168.23) by their MS spectra (**C**), which should be biosynthesized from zeaxanthin with carotenoid cleavage dioxygenase 2 (CCD2) (3, 17), as shown in Figure 1. It is thus likely that these two volatile compounds are present not only in saffron but also in freesia yellow flowers.

### Chemical Changes of Apocarotenoids Crocetin Glycosides in Saffron

**Figure 4:**
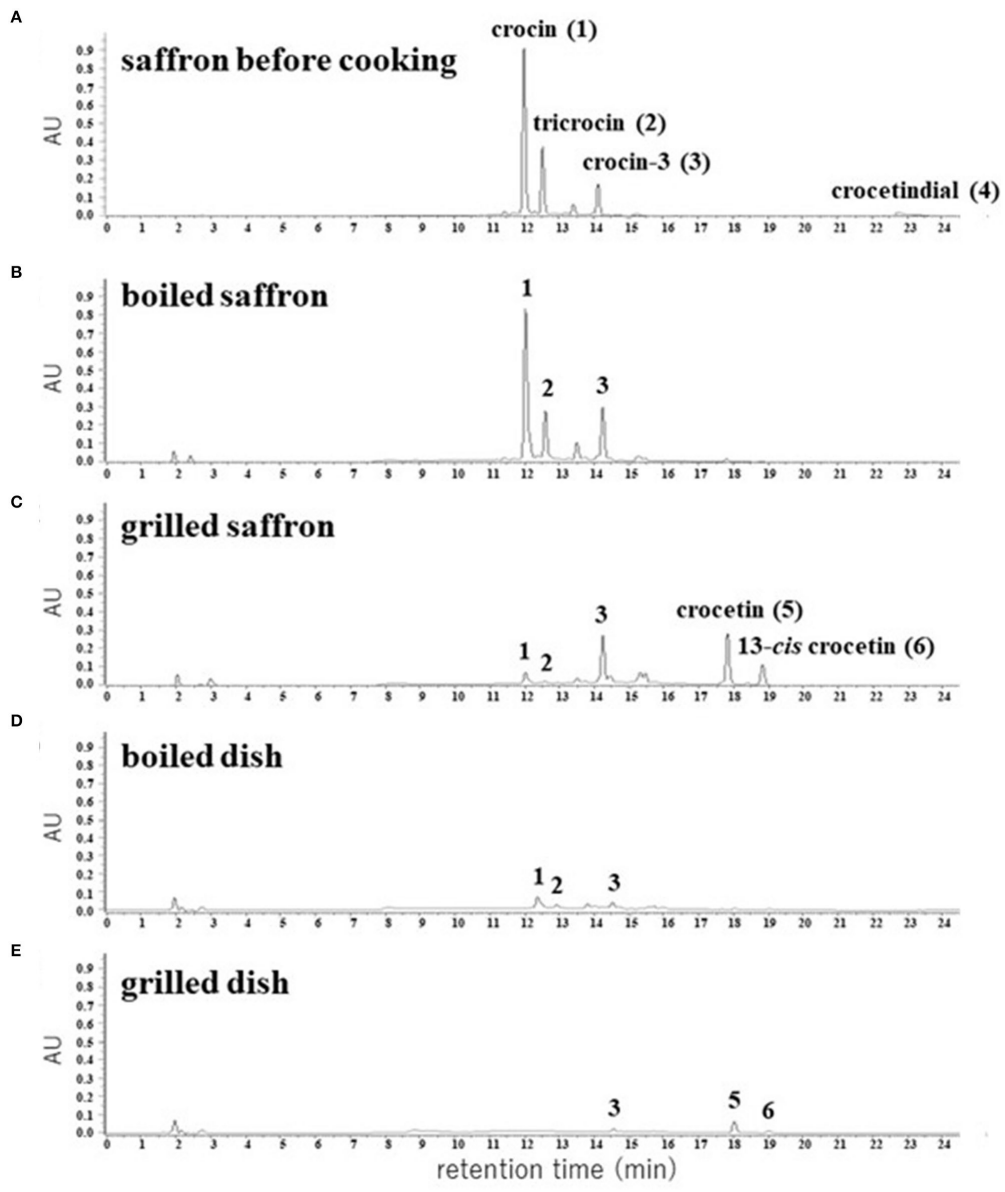
HPLC chromatograms of apocarotenoids extracted from the dried pistils of saffron **(A)** and their cooked materials **(B–E)**. The detection wavelength was 440 nm.

**Figure 5:**
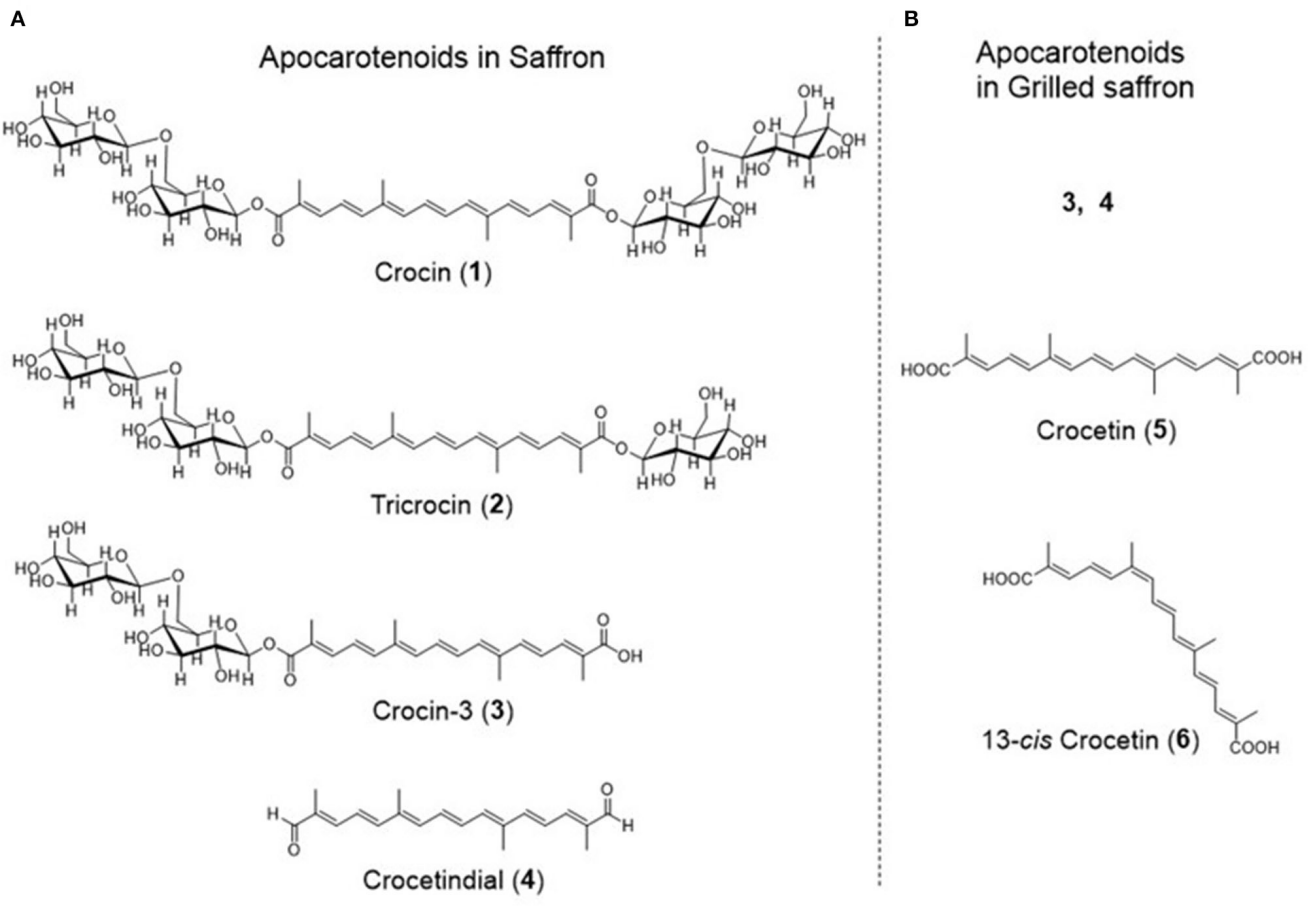
The structures of apocarotenoids in saffron **(A)** and grilled saffron **(B)**.

We extracted apocarotenoids from saffron (dried; 0.08 g) as well as its boiled and grilled samples by the cooking models, and analyzed them by HPLC-DAD (Figures 4A–C). Six apocarotenoids corresponding to peaks **1**–**4** and peaks **5**, **6** were isolated from saffron and the grilled saffron, respectively, by the methods described in Material and methods, and subjected to HR-ESI-MS, ^1^H and ^13^C NMR analyses. The results, **1**–**6** were identified as crocin (2), tricrocin (18), crocin-3 (18), crocetindial (19), crocetin (20), and 13-*cis* crocetin (21), respectively (their ^1^H and ^13^C NMR spectra of **1**–**6** are shown in Supplementary Figures S1–S12). The chemical structures of **1**–**6** are exhibited in Figure 5.

The predominant apocarotenoid **1** and the other main apocarotenoids **2** and **3** showed the same UV-Vis spectra that possess λmax at 440 nm depending on the same aglycone, and minor apocarotenoids **4**–**6** also exhibited λmax around 440 nm (Supplementary Figure S13). Thus, we calculated the peak areas of individual apocarotenoids (**1**–**6**) and total apocarotenoids (**1** + **2** + **3** + **4** + **5** **+** **6**) at 440 nm in saffron, the boiled saffron, and the grilled saffron in HPLC-DAD analyses, as listed in Table 1. In the boiled cooking model of saffron, the amount of crocin (**1**), tricrocin (**2**), and crocin-3 (**3**) almost did not change, while crocetindial (**4**) disappeared, presumably due to its decomposition or a chemical reaction to other compounds. In the grilled cooking model of saffron, the amount of crocin (**1**) and tricrocin (**2**) decreased, the amount of crocin-3 (**3**) slightly increased, and crocetin (**5**) and 13-*cis* crocetin (**6**) appeared (Figure 4C). In addition, the total amount of apocarotenoids decreased to about 1/3 (Table 1). Supplementary Figure S18 also shows that the purified crocin (**1**) and crocetin (**2**) were partially changed into crocetin (**5**) and 13-*cis* crocetin (**6**), and into **6**, respectively, at 180°C for 5 min. These observations indicated that hydrolysis of the ester linkage between glucose—the crocetin aglycone occurred, presumably by conversion from crocin and tricrocin to crocin-3 and further to crocetin, along with the subsequent *trans* to 13-*cis* isomerization of a part of crocetin. It is also likely that the degradation of the apocarotenoids occurred through strong heat cooking like grilling.

**Table 1:** HPLC peak areas (mV × s) of apocarotenoids in cooked saffron at 440 nm.

**Cooking**\**apocarotenoid**	**Crocin** **(1)**	**Tricrocin** **(2)**	**Crocin-3** **(3)**	**Crocetindial** **(4)**	**Crocetin** **(5)**	**13-*cis* crocetin** **(6)**	**Total apocarotenoid peak areas** **(1 + 2 + 3 + 4 + 5 + 6)**
Saffron (before cooking)	3.2 ×10^6^	1.1 ×10^6^	6.0 ×10^5^	3.5 ×10^4^	N.D.	N.D.	5.0 ×10^6^
Boiled saffron	2.6 ×10^6^	8.1 ×10^5^	5.6 ×10^5^	N.D.	N.D.	N.D.	4.4 ×10^6^
Boiled saffron/saffron	81%	74%	93%	0%			88%
Grilled saffron	2.1 ×10^6^	2.9 ×10^5^	7.5 ×10^5^	N.D.	6.3 ×10^5^	3.2 ×10^5^	1.8 ×10^6^
Grilled saffron/saffron	66%	26%	125%	0%			36%
Boiled dish	2.6 ×10^5^	4.5 ×10^4^	6.7 ×10^4^	N.D.	N.D.	N.D.	9.8 ×10^5^
Boiled dish/saffron	8.1%	4.1%	11%	0%			20%
Grilled dish	N.D.	N.D.	3.8 ×10^4^	N.D.	4.0 ×10^5^	3.0 ×10^4^	4.0 ×10^5^
Grilled dish/saffron	0%	0%	6.3%	0%			8.0%

In order to confirm whether the same chemical changes occur in actual cooking, saffron was mixed with rice and a typical boiling and grilling cooking process to prepare dishes was performed. The apocarotenoids contained in the dishes were extracted and subjected to HPLC-DAD analyses (Figures 4D,E; Table 1). These results apparently showed that the same chemical changes occurred in the actual cooking. However, the total peak area of detected apocarotenoids in both dishes has decreased to about 1/5-−1/10. It is currently under investigation what happens to such disappearing apocarotenoids.

### Identification of Apocarotenoids Crocetin Glycosides in Freesia

The apocarotenoids in freesia “f2” flowers (**7** and **8**) were isolated through chromatographic methods as described in Material and method.

**Figure 6:**
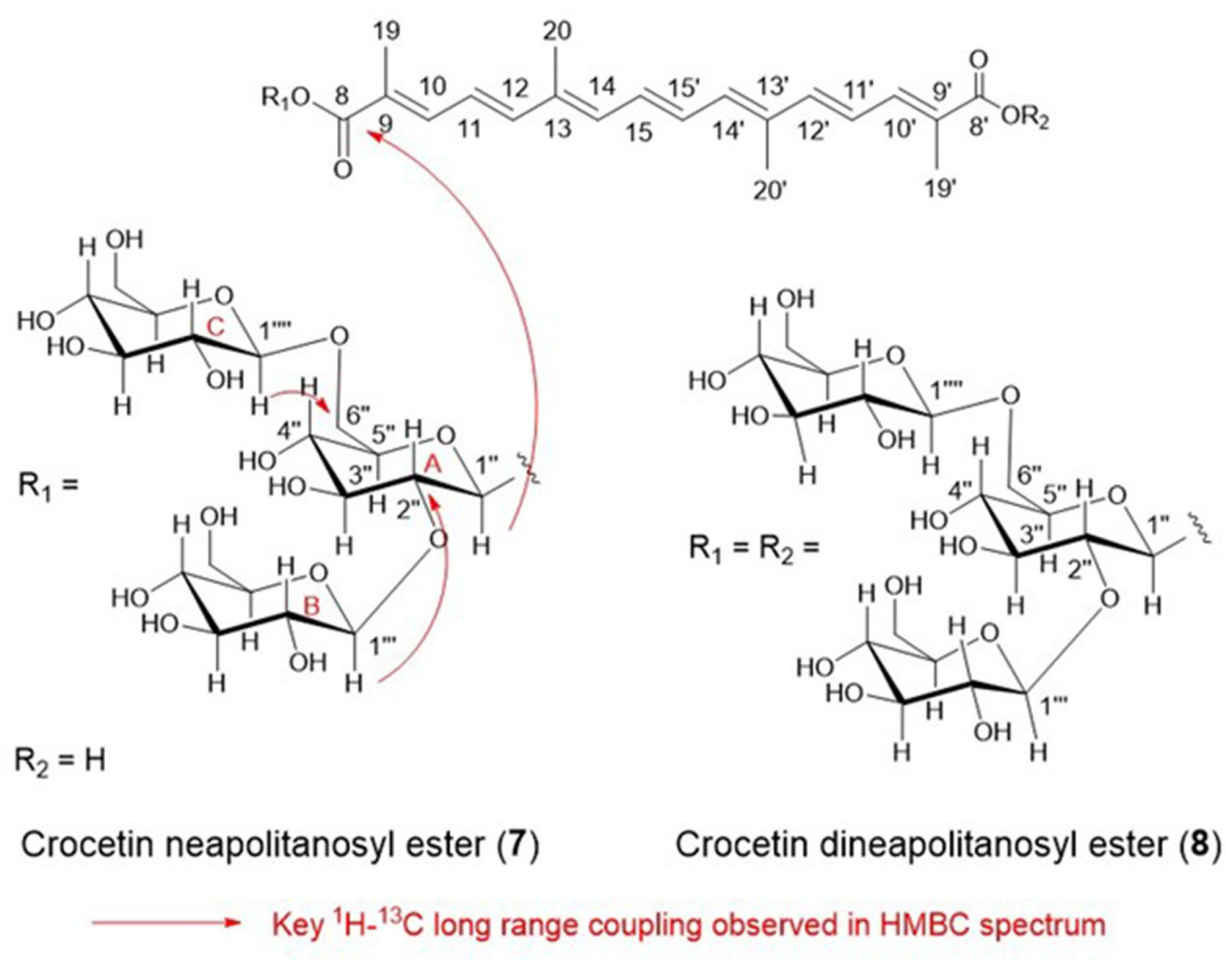
The structures of apocarotenoids in freesia.

The structures of **7** were analyzed by HR-ESI-MS and 1D (^1^H and ^13^C) and 2D (^1^H-^1^H DQF COSY, HMQC, and HMBC) NMR spectra. HR-ESI-MS analysis of **7** showed (M + Na)^+^ at *m/z* 837.31834, and the molecular formula of **7** was determined as C_38_H_54_O_19_ [(M + Na)^+^ C_38_H_54_O_19_Na calcd. for 837.31570 (Δ3.16 ppm)]. The molecular formula of **7** was identical to that of tricrocin, and the ^1^H and ^13^C NMR spectra of **7** in CD_3_OD (Supplementary Figures S13, S14) were closely related to tricrocin but not identical. The carotenoid aglycone of **7** proved to be identical to that of tricrocin (=crocetin) by comparison the ^1^H and ^13^C NMR with those of crocetin. Since three anomeric signals [δ_H_ 4.32 (*J* = 7.6 Hz), δ_H_ 4.59 (*J* = 7.8 Hz), δ_H_ 5.65 (*J* = 7.8 Hz)] with β configuration were observed in the ^1^H NMR spectrum of **7** and since the molecular formula of **7** (C_38_H_54_O_19_) was [crocetin (C_20_H_24_O_4_) + 3 hexose (C_18_H_36_O_18_)−3H_2_O], **7** was proposed to possess a structure in which 3 β-hexoses were bound to crocetin. To determine the type of hexose, **7** (5.0 mg) was hydrolyzed in 2 M HCl (5 ml) under reflux for 2 h, and the hydrolysate was partitioned with EtOAc (5 ml) twice. The H_2_O layer containing hexose was concentrated to dryness (2.0 mg), and analyzed by ^1^H and ^13^C NMR in D_2_O, and [α]_D_ [+68.3° (*c* 0.1, H_2_O)]. These data clearly proved that hexoses in **7** were all D-glucose. The linkages of 3 β-D-glucose to crocetin in **7** were analyzed by ^1^H-^1^H DQF COSY, HSQC, and HMBC, and the linkages among glucoses were proved to be glucoseB(1 → 2)glucoseA and glucoseC(1 → 6)glucoseA and the ester linkage of glucoseA to crocetin was proved by the key ^1^H-^13^C long-range couplings observed in HMBC spectrum (from H-1”' (δ_H_ 4.59) to C-2” (δ_C_ 82.4), from H-1”” (δ_H_ 4.32) to C-6” (δ _C_ 69.4), and from H-1” (δ _H_ 5.65) to C-8 (δ _C_ 168.1) as shown in Figure 6. From these observations, the structure of **7** was determined to be crocetin neapolitanosyl ester (Figure 6).

The production of **7** by artificial manufacturing processing was reported in two previous studies (22, 23), while this is the first report finding **7** in natural flowers.

HR-ESI-MS analysis of **8** showed (M-H)^−^ at *m/z* 1299.47367, and the molecular formula of **8** was determined as C_56_H_84_O_34_ [(M-H)^−^ C_56_H_84_O_33_ calcd. for 1299.47657 (Δ 2.23 ppm), and this formula was identical with that of crocetin dineapolitanosyl ester which was reported as a minor yellow pigment produced by *Crocus sativus*. Thus, we compared ^1^H and ^13^C NMR spectrum of **8** (Supplementary Figures S15, S16) with those of reported crocetin dineapolitanosyl ester (2), and identified **8** as crocetin dineapolitanosyl ester (Figure 6).

### Chemical Changes of Apocarotenoids Crocetin Glycosides in Freesia

**Figure 7:**
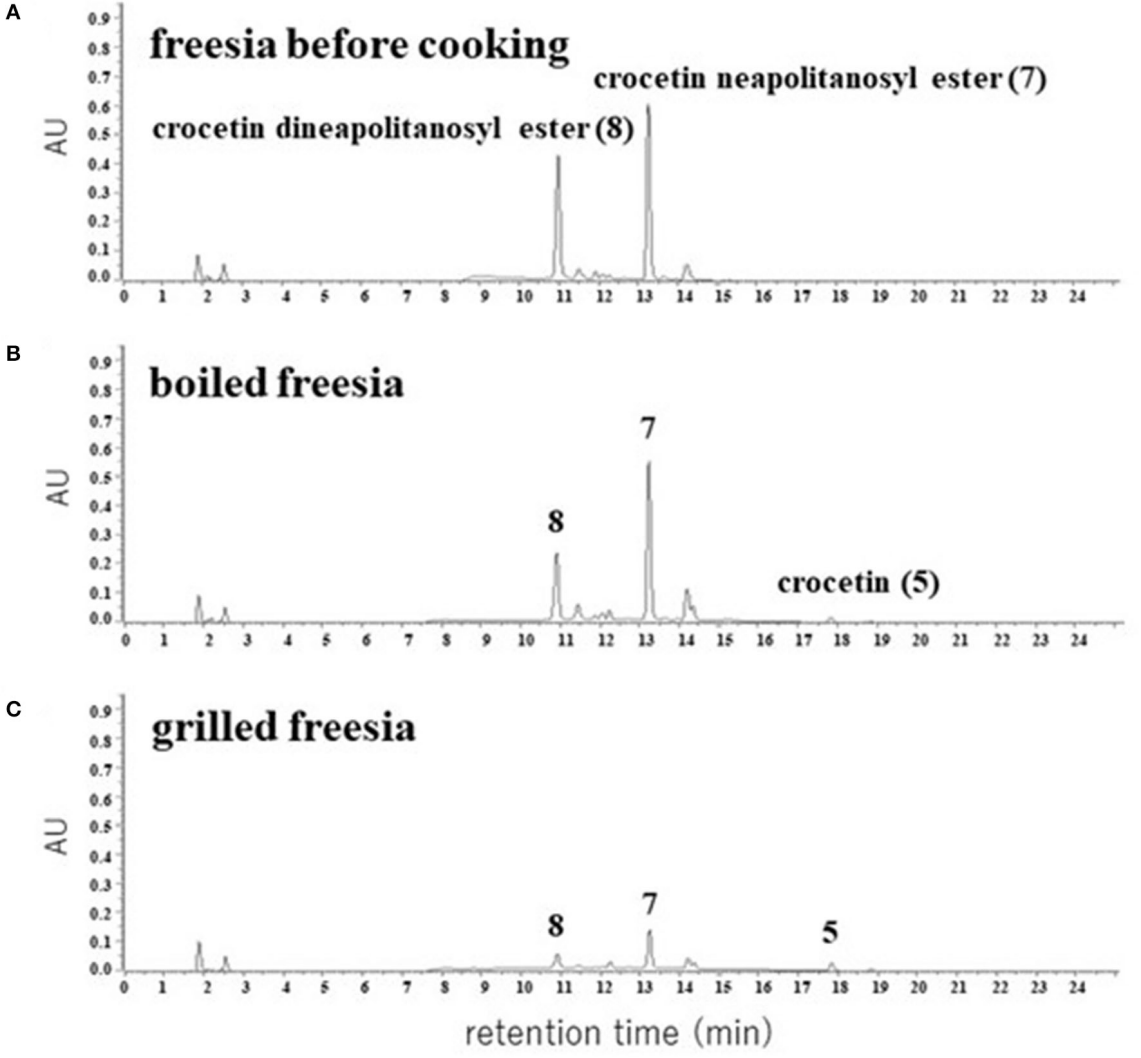
HPLC chromatograms of apocarotenoids extracted from the yellow petals of freesia “Kayak” **(A)** and their cooked materials **(B,C)**. The detection wavelength was 440 nm.

We extracted apocarotenoids from the yellow petals of freesia cultivar “Kayak” [1.8 g (fresh weight)] as well as their boiled and grilled samples by cooking models, and analyzed them by HPLC-DAD (Figure 7). Figure 7A (Table 2) revealed that the petals of freesia “Kayak,” like the case of “f2,” possessed the dineapolitanosyl ester and (mono)neapolitanosyl ester of crocetin dominantly, which were 48 and 52% of whole apocarotenoids when calculated by peak areas at 440 nm, respectively. In the boiled cooking model in freesia, the amount of crocetin dineapolitanosyl ester was reduced to 59% and a small amount of crocetin appeared, while the level of crocetin (mono)neapolitanosyl ester was not changed (Table 2; Figure 7B). In the grilled cooking model in freesia, the amounts of crocetin dineapolitanosyl ester and crocetin neapolitanosyl ester were reduced to 11 and 23%, respectively, and a small amount of crocetin also appeared (Table 2; Figure 7C). These results indicated that hydrolysis of the ester linkage between glucose—the crocetin aglycone occurred in both cooking models, presumably by conversion from crocetin dineapolitanosyl ester to crocetin neapolitanosyl ester and further to crocetin. The degradation of the apocarotenoids is likely to occur specifically through strong heat cooking like grilling.

**Table 2:** HPLC peak areas (mV × s) of apocarotenoids in cooked freesia yellow flowers at 440 nm.

**Cooking**\**apocarotenoid**	**Crocetin dineapolitanosyl ester** **(8)**	**Crocetin neapolitanosyl ester** **(7)**	**Crocetin** **(5)**	**Total apocarotenoid peak areas** **(8 + 7 + 5)**
Freesia (before cooking)	5.6 ×10^6^	6.1 ×10^6^	N.D.	1.2 ×10^7^
Boiled freesia	3.3 ×10^6^	5.9 ×10^6^	2.3 ×10^5^	9.2 ×10^6^
Boiled freesia/freesia	59%	97%		84%
Grilled freesia	5.9 ×10^5^	1.4 ×10^6^	1.4 ×10^5^	2.1 ×10^6^
Grilled freeisia/freesia	11%	23%		18%

### Significance of This Study on Freesia

We also extracted and analyzed apocarotenoids from the flowers of freesia cultivar “Aladin” in the same way, and found that freesia “Aladin” possessed the dineapolitanosyl ester and (mono)neapolitanosyl ester of crocetin dominantly, like “f2” and “Kayak.”

This study revealed that the main pigments in the yellow flowers of freesia (*Freesia* x *hybrida*) were unique apocarotenoids, crocetin (mono)neapolitanosyl ester, and crocetin dineapolitanosyl ester. Plants that include the (mono)neapolitanosyl ester of crocetin have not been reported so far. The stigmas of *Crocus sativus* (saffron) and the fruits of *Gardenia jasminoides*, retain crocin predominantly (1, 2). Recently, fully open mature flowers of *G. jasminoides* were shown to accumulate crocin-3 and crocetin as main apocarotenoids (24). These findings suggest that, among plants producing crocetin glycosides, only freesia retains UDP-glucose transferase(s) [UGT(s)] that has activity sufficient for constructing the neapolitanosyl group composed of three molecules of glucose, as shown in Figure 1B. The microscopy image of the petal of cultivar “f2” showed that such yellow apocarotenoids accumulate not in plastids (chromoplasts) but vacuoles (Figure 2G).

In addition, we demonstrated that the freesia apocarotenoids can keep yellow color after bread and rice cooking, as shown in Figures 2D,F. This study opens the feasibility of freesia yellow flowers as new edible flowers with beneficial functions for human health.

### ^1^O_2_-Quenching Activities of Apocarotenoids

Many carotenoids were reported to possess ^1^O_2_-quenching activity depending on their olefin structures (14). Although such activity of crocin (**1**) was reported (25), ^1^O_2_-quenching activity of **2**–**8** has not been reported so far. Thus, we examined these ^1^O_2_-quenching activities and proved that **1**–**8** possessed almost equivalent moderate ^1^O_2_-quenching activity (IC_50_ 24–64 μM), presumably due to their same conjugation number (=9) (Table 3). On the other hand, apocarotenoids **1**–**8** did not show DPPH radical scavenging activities and lipid peroxidation-inhibiting activities even at 100 μM, according to our experiment.

**Table 3:** ^1^O_2_-quenching activities of apocarotenoids and astaxanthin.

**Compound**	**IC_**50**_ (μM)**
Crocin (**1**)	48 ± 0.4
Tricrocin (**2**)	46 ± 3.5
Crocin-3 (**3**)	64 ± 9.1
Crocetindial (**4**)	24 ± 1.9
Crocetin (**5**)	54 ± 7.4
13-*cis* crocetin (**6**)	54 ± 8.5
Crocetin neapolitanosyl ester (**7**)	56 ± 2.7
Crocetin dineapolitanosyl ester (**8**)	64 ± 6.3
Astaxanthin (control)	1.4 ± 0.1

## Conclusion

In this study, we first reported chemical changes of apocarotenoids in saffron and freesia through cooking models as well as identification of unique apocarotenoids in freesia flowers. We also evaluated the ^1^O_2_-quenching activities of apocarotenoids in saffron and freesia.

## Data Availability Statement

The datasets presented in this study can be found in online repositories. The names of the repository/repositories and accession number(s) can be found in the article/Supplementary Material.

## Author Contributions

This research was conceived and supervised by KS and NM. Laboratory experiments were done by KS, YS, SS, CT, MA, SU, YU, J-iH, and II. Field experiments were carried out by AK, MK, and MM. Data were analyzed by KS, J-iH, MT, and NM. Pictures were recorded by NM. The manuscript was written by KS and NM. All authors contributed to the article and approved the submitted version.

## Conflict of Interest

KS, II, and NM were employed by CaroProTech Corporation. The remaining authors declare that the research was conducted in the absence of any commercial or financial relationships that could be construed as a potential conflict of interest.

## Publisher's Note

All claims expressed in this article are solely those of the authors and do not necessarily represent those of their affiliated organizations, or those of the publisher, the editors and the reviewers. Any product that may be evaluated in this article, or claim that may be made by its manufacturer, is not guaranteed or endorsed by the publisher.
