# Supplementary material for: Changes of Crocin and Other Crocetin Glycosides in Saffron Through Cooking Models, and Discovery of Rare Crocetin Glycosides in the Yellow Flowers of Freesia Hybrida
Source: Front Nutr. 2022 Jul 14;9:885412. doi: 10.3389/fnut.2022.885412 (PMC9331930; doi:10.3389/fnut.2022.885412)
Supplement: Supplementary file 1 [file Presentation_1.pdf]

SUPPLEMENTARY MATERIAL

Changes of crocin and other crocetin glycosides in saffron through cooking models, and discovery of rare crocetin glycosides in the yellow flowers of *Freesia hybrida*.

Kazutoshi Shindo, Yuka Sakemi, Saki Shimode, Chiharu Takagi, Yohei Uwagaki, Jun-ichiro Hattan, Miu Akao, Shiori Usui, Ayako Kiyokawa, Masako Komaki, Minoru Murahama, Miho Takemura, Isamu Ishikawa, and Norihiko Misawa

Graphic Abstract

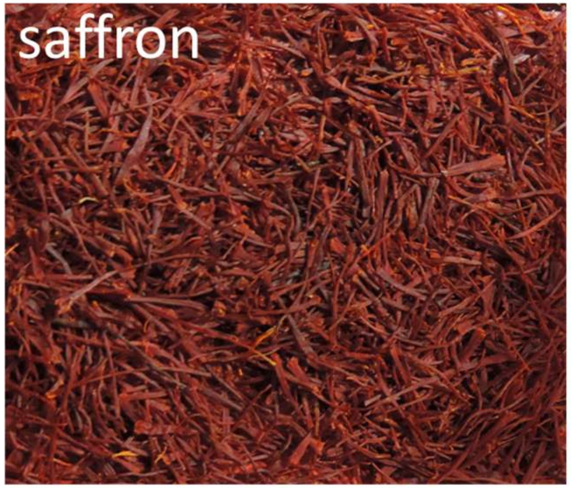

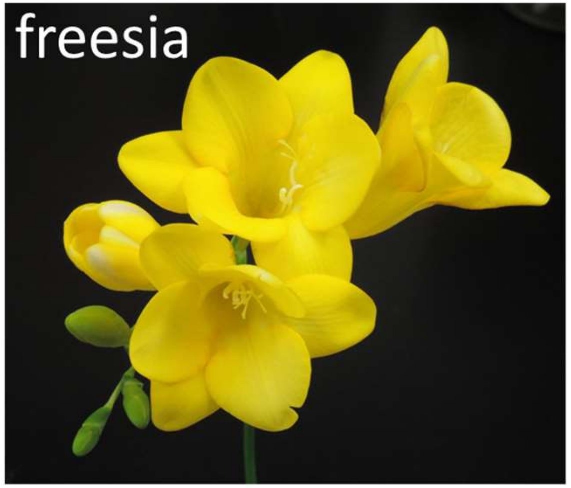

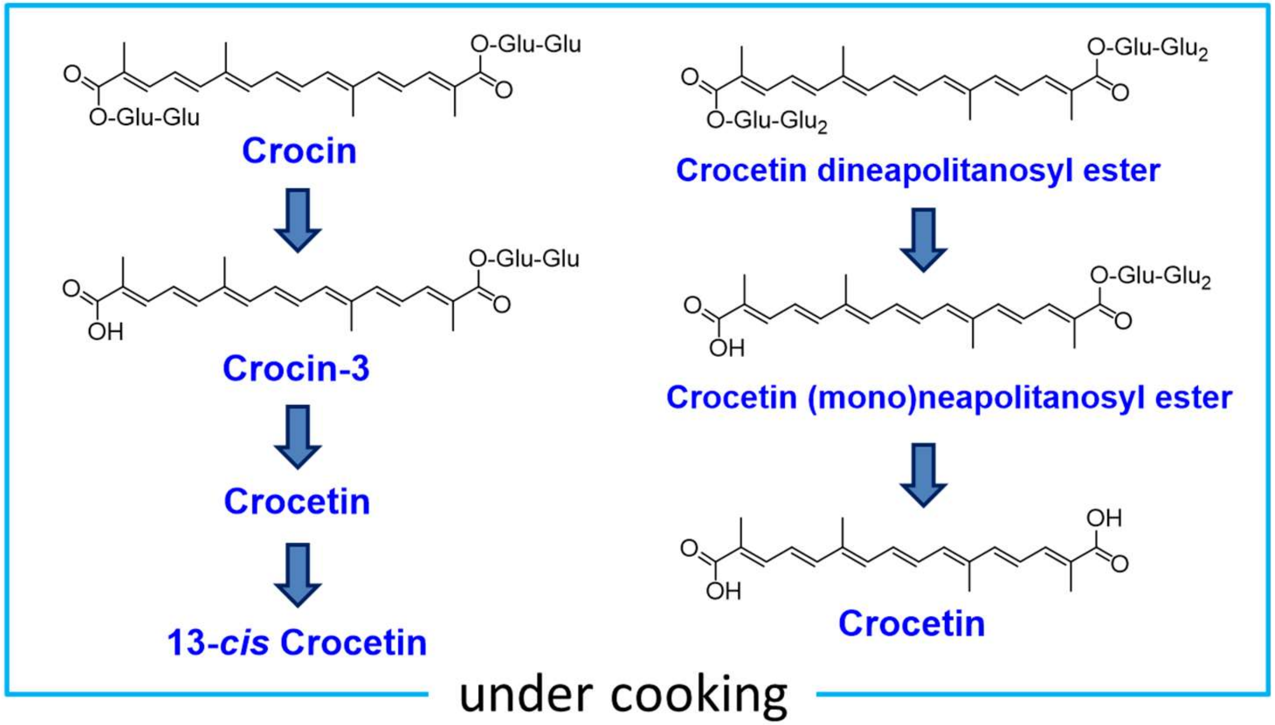

Supplementary Figures

Figure S1. ^{1}H NMR spectrum of crocin (**1**) in CD_3OD.
Figure S2. ^{13}C NMR spectrum of crocin (**1**) in CD_3OD.
Figure S3. ^{1}H NMR spectrum of tricrocin (**2**) in CD_3OD.
Figure S4. ^{13}C NMR spectrum of tricrocin (**2**) in CD_3OD.
Figure S5. ^{1}H NMR spectrum of crocin-3 (**3**) in CD_3OD.
Figure S6. ^{13}C NMR spectrum of crocin-3 (**3**) in CD_3OD.
Figure S7. ^{1}H NMR spectrum of crocetindial (**4**) in $CDCl_3$.
Figure S8. ^{13}C NMR spectrum of crocetidinal (**4**) in $CDCl_3$.
Figure S9. ^{1}H NMR spectrum of crocetin (**5**) in CD_3OD.
Figure S10. ^{13}C NMR spectrum of crocetin (**5**) in CD_3OD.
Figure S11. ^{1}H NMR spectrum of 13-*cis* crocetin (**6**) in CD_3OD.
Figure S12. ^{13}C NMR spectrum of 13-*cis* crocetin (**6**) in CD_3OD.
Figure S13. ^{1}H NMR spectrum of crocetin neapolitanosyl ester (**7**) in CD_3OD.
Figure S14. ^{13}C NMR spectrum of crocetin neapolitanosyl ester (**7**) in CD_3OD.
Figure S15. ^{1}H NMR spectrum of crocetin dineapolitanosyl ester (**8**) in CD_3OD-D_2O (1:1).
Figure S16. ^{13}C NMR spectrum of crocetin dineapolitanosyl ester (**8**) in CD_3OD-D_2O (1:1).
Figure S17. UV-Vis spectra of crocin (**1**), tricrocin (**2**), crocin-3 (**3**), crocetindial (**4**), crocetin (**5**), and 13-*cis* crocetin (**6**) in HPLC-DAD analysis.
Figure S18. Changes of crocin (**1**) and crocetin (**5**) through the grilled cooking model.

Figure S1. ^1H NMR spectrum of crocin (**1**) in CD_3OD.

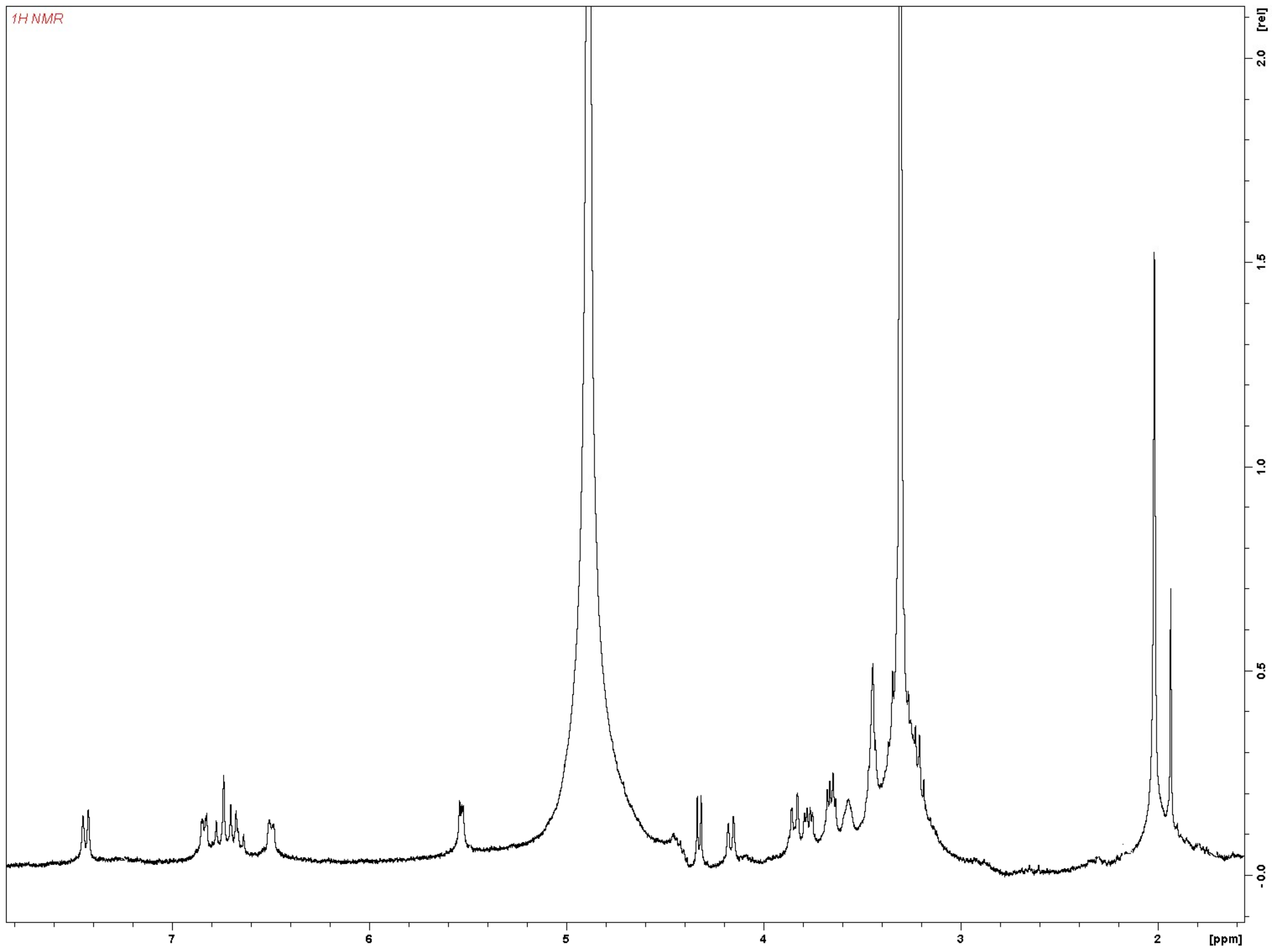

Figure S2. ^{13}C NMR spectrum of crocin (**1**) in CD_3OD.

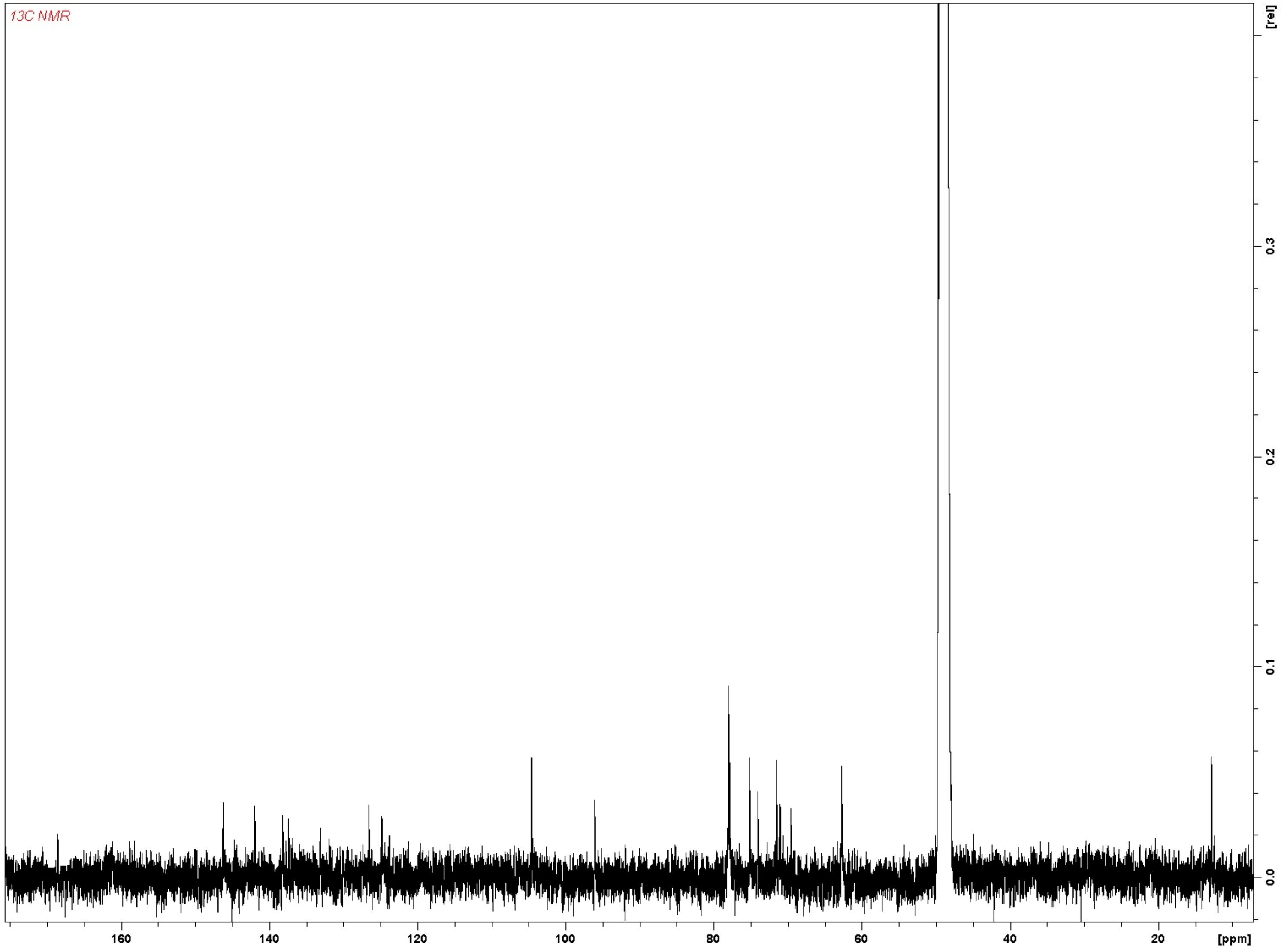

Figure S3. ^{1}H NMR spectrum of tricrocin (**2**) in CD_3OD.

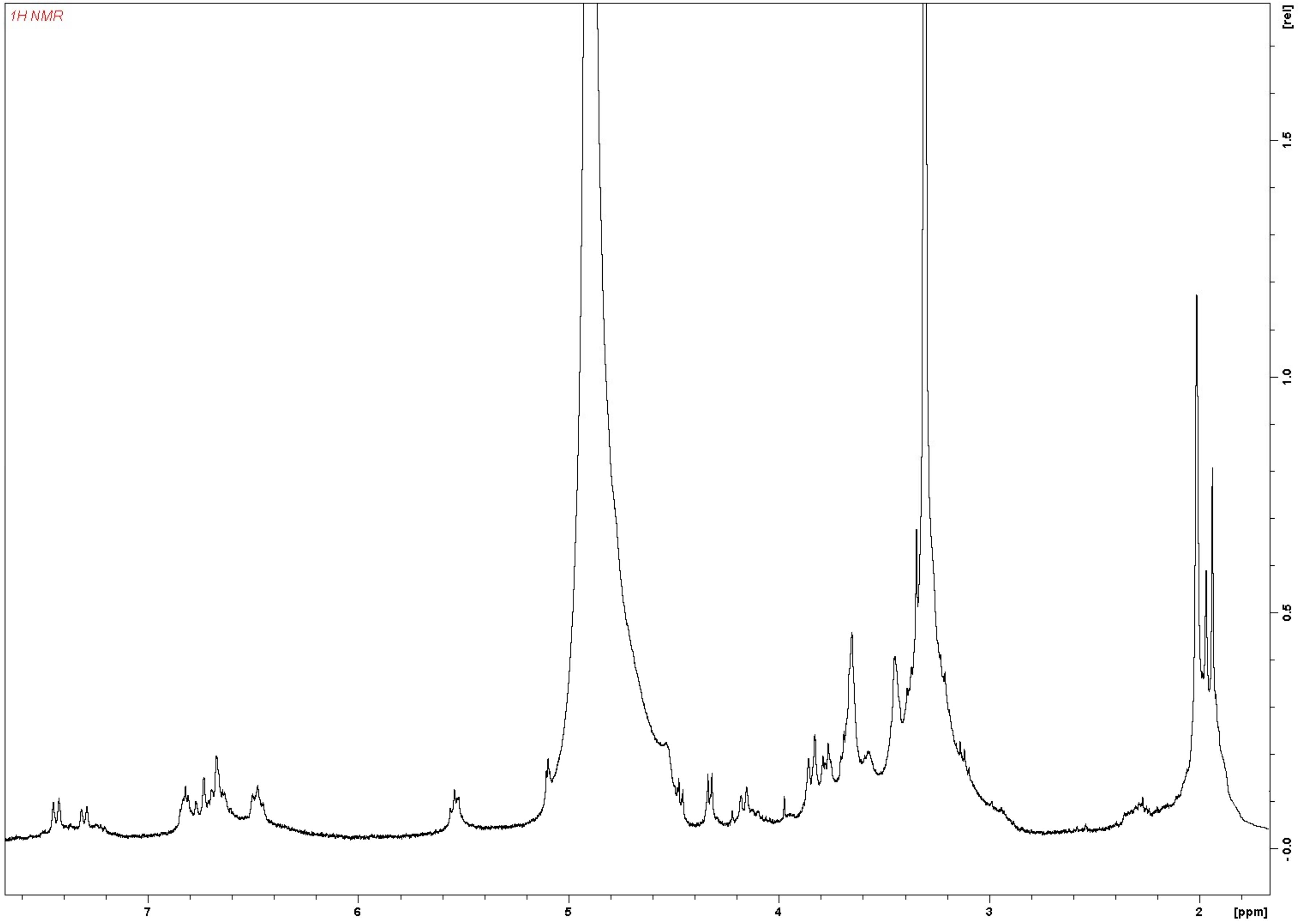

Figure S4. ^{13}C NMR spectrum of tricrocin (**2**) in CD_3OD.

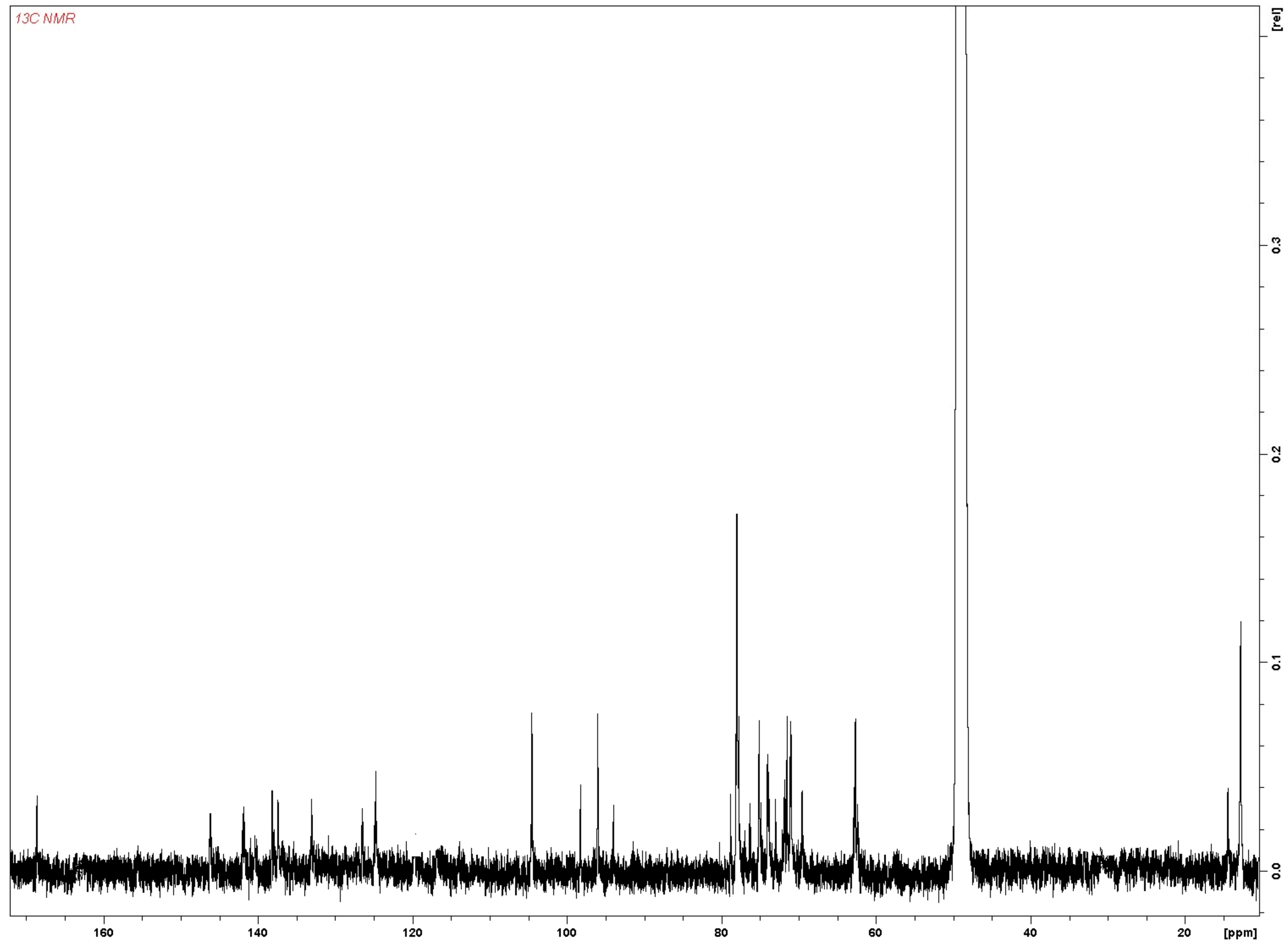

Figure S5. ^{1}H NMR spectrum of crocin-3 (**3**) in CD_3OD.

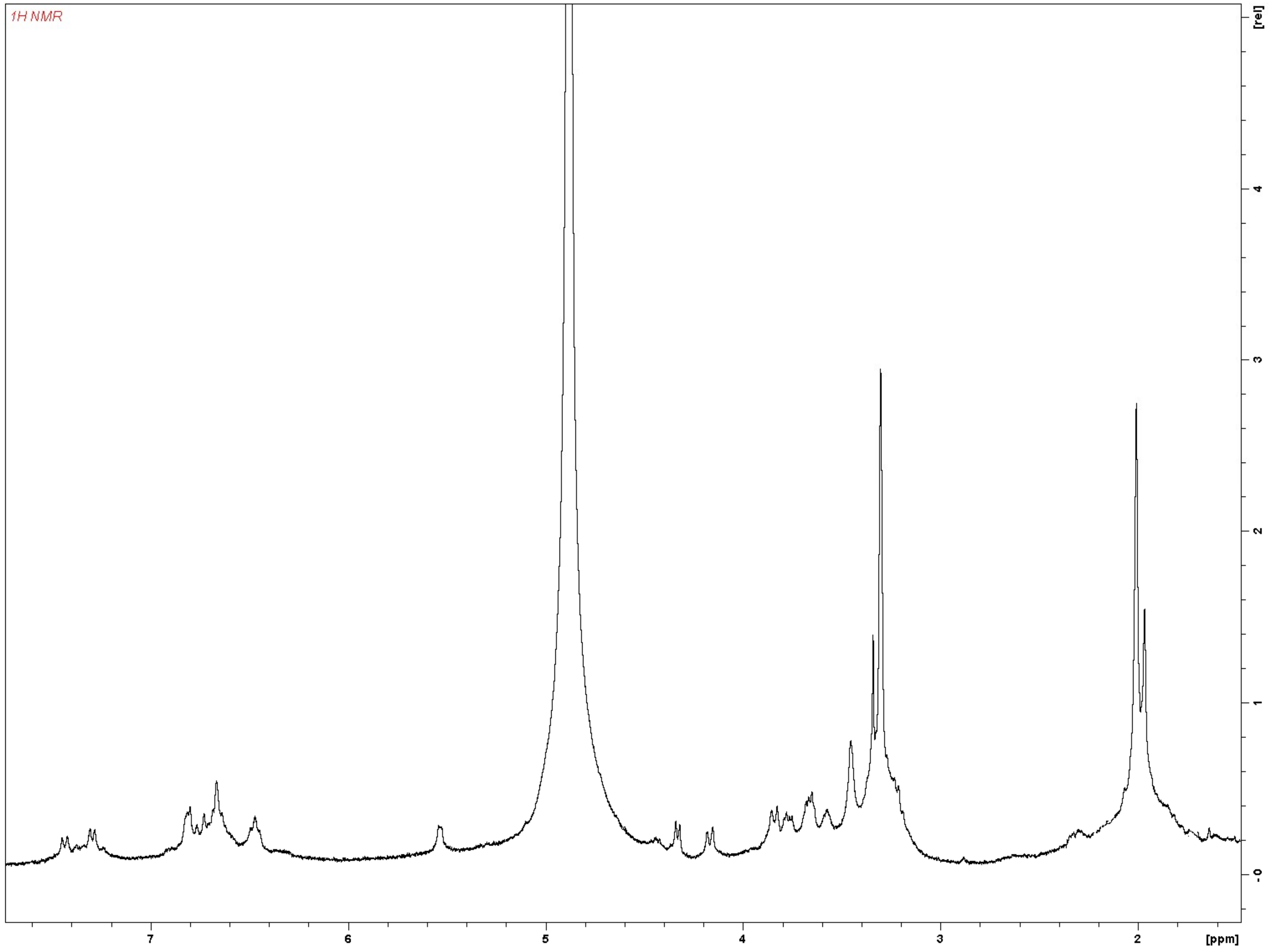

Figure S6. ^{13}C NMR spectrum of crocin-3 (**3**) in CD_3OD.

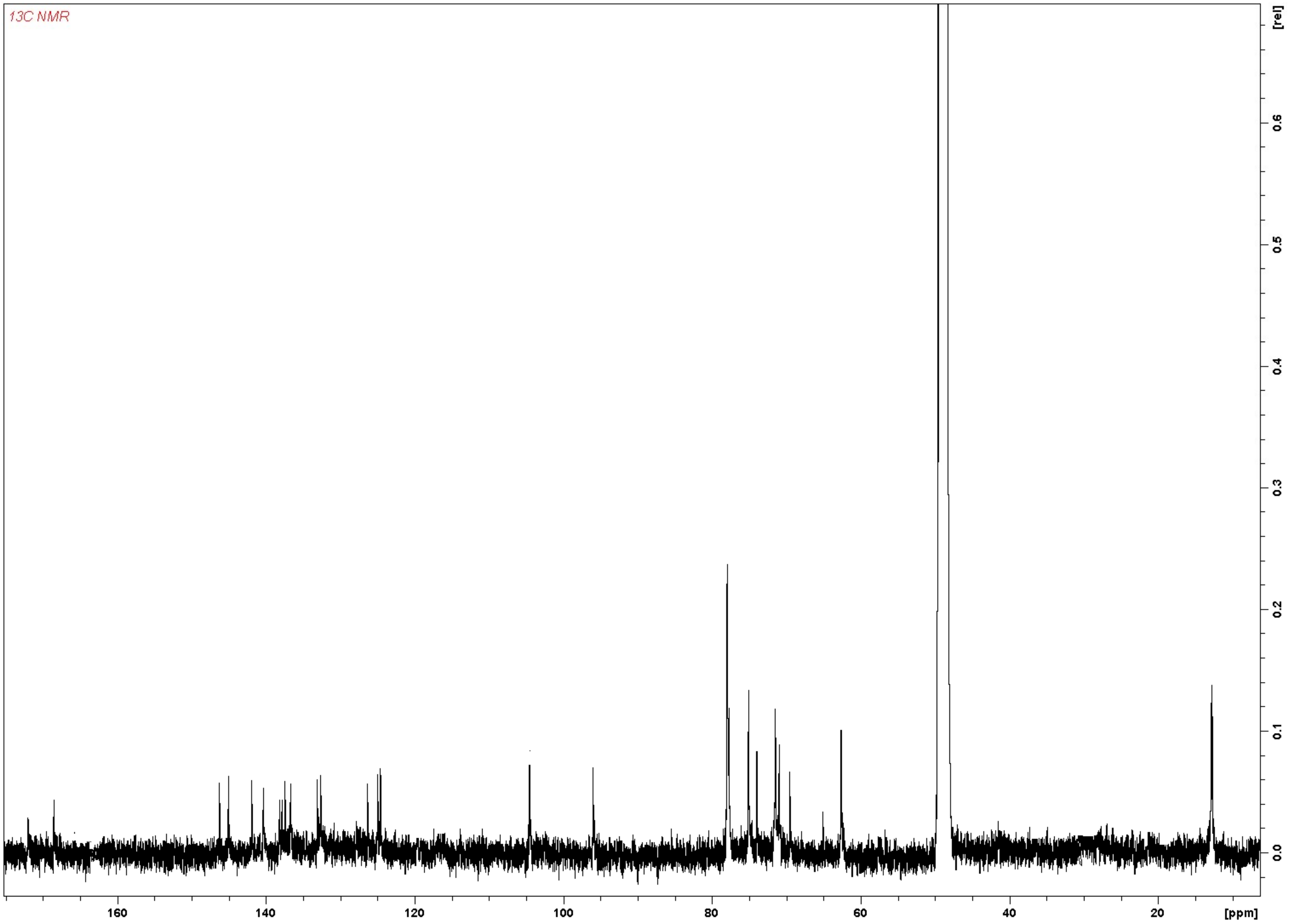

Figure S7. ^1H NMR spectrum of crocetindial (**4**) in $CDCl_3$.

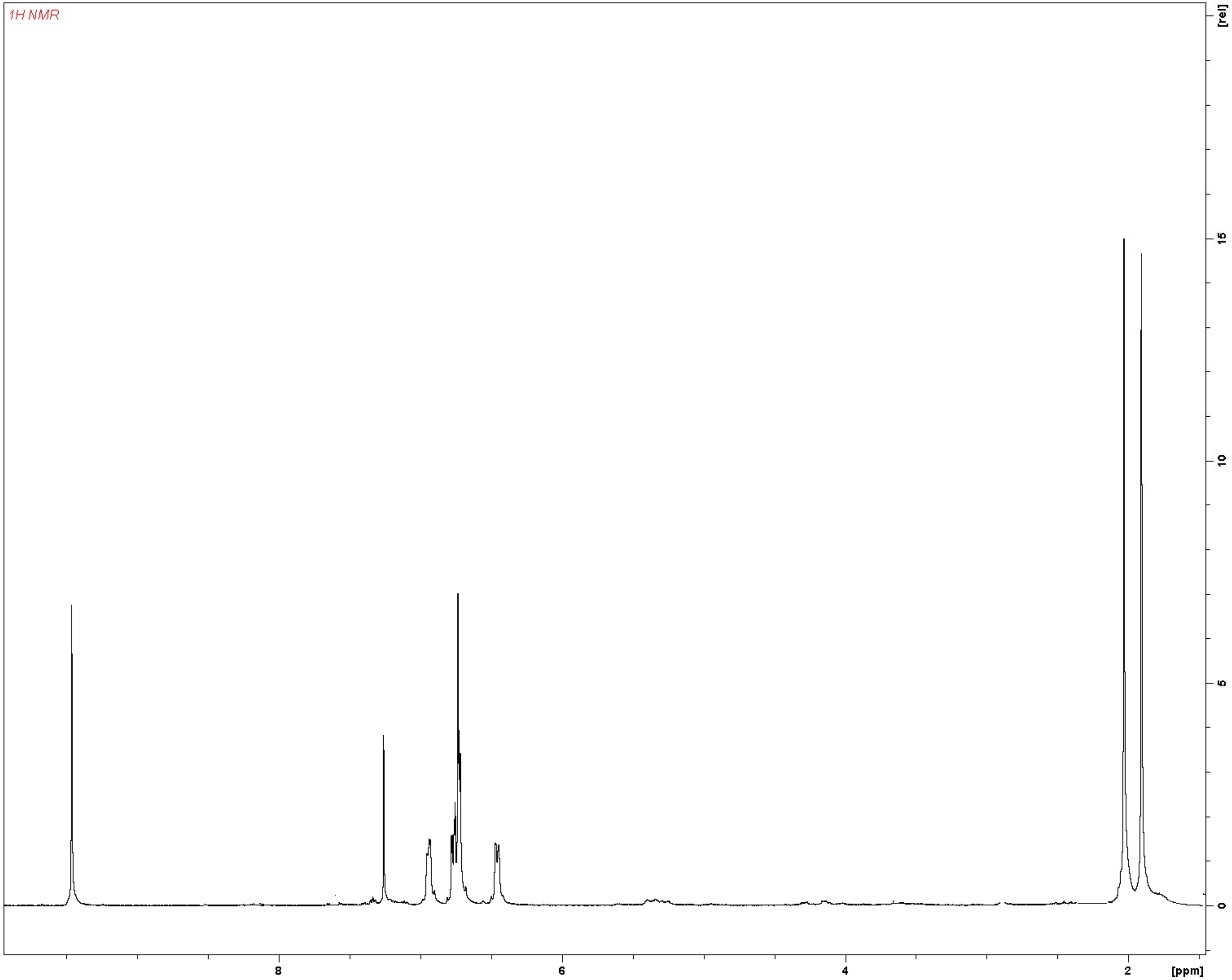

Figure S8. ^{13}C NMR spectrum of crocetidinal (**4**) in $CDCl_3$.

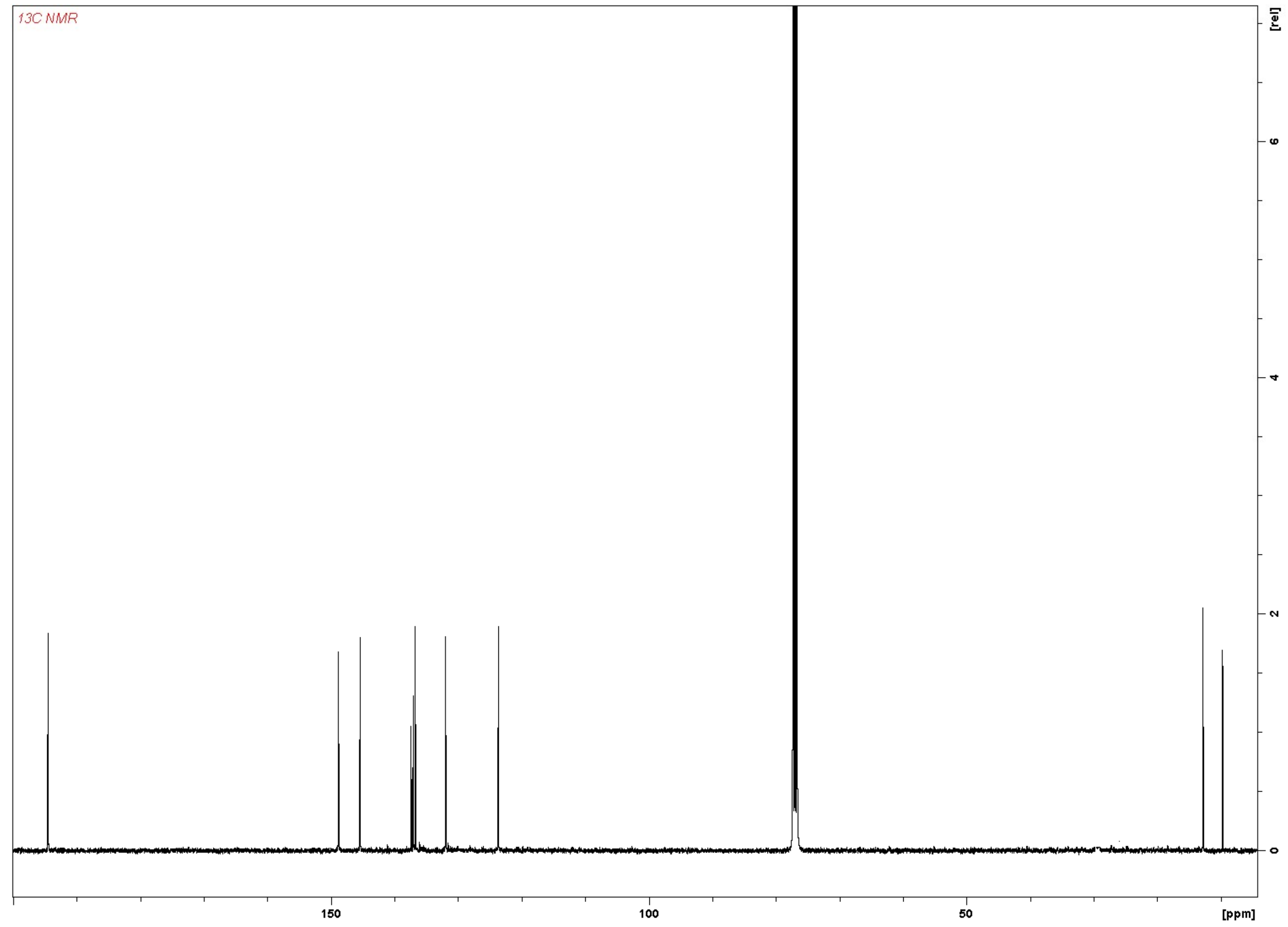

Figure S9. ^{1}H NMR spectrum of crocetin (**5**) in CD_3OD.

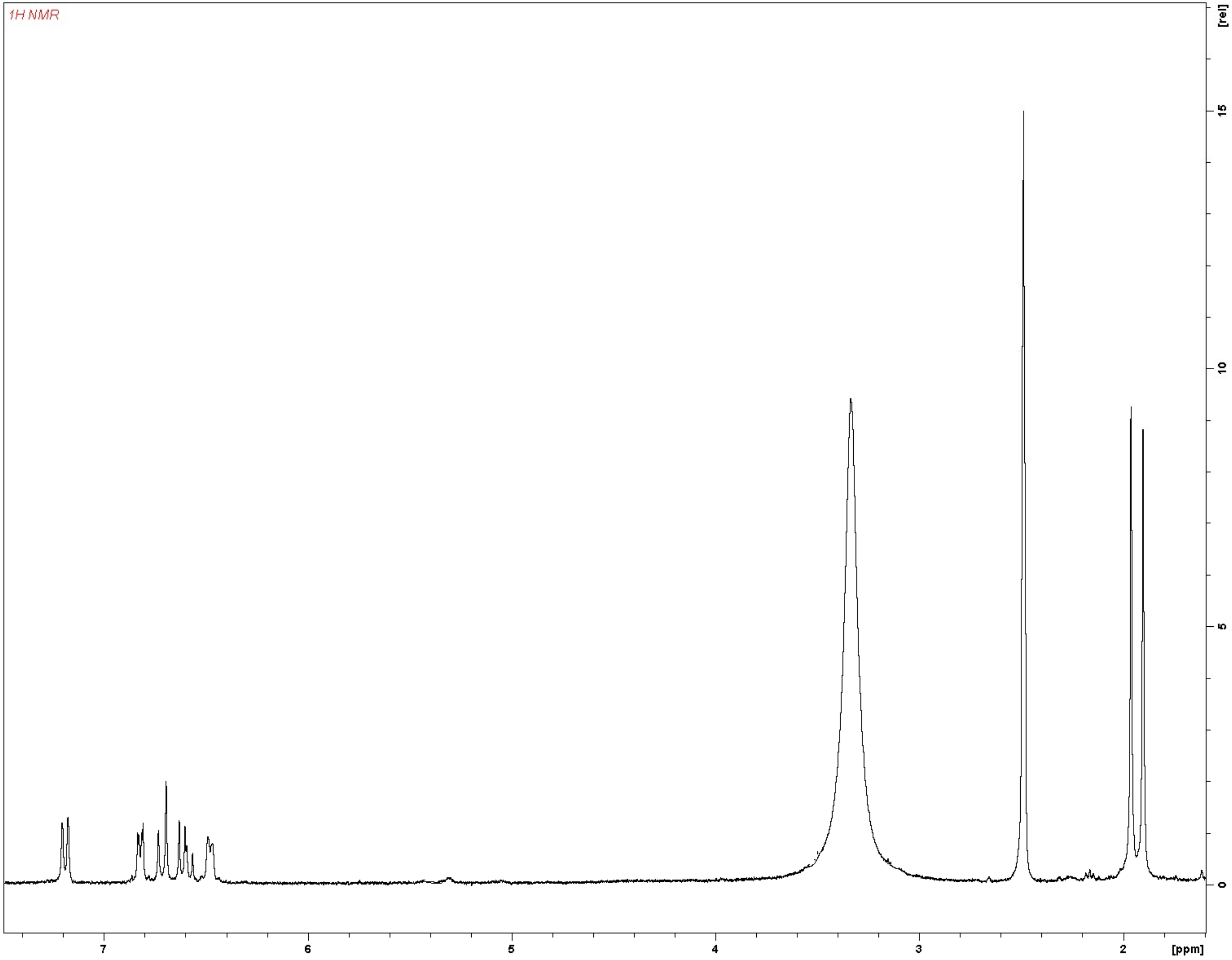

Figure S10. ^{13}C NMR spectrum of crocetin (**5**) in CD_3OD.

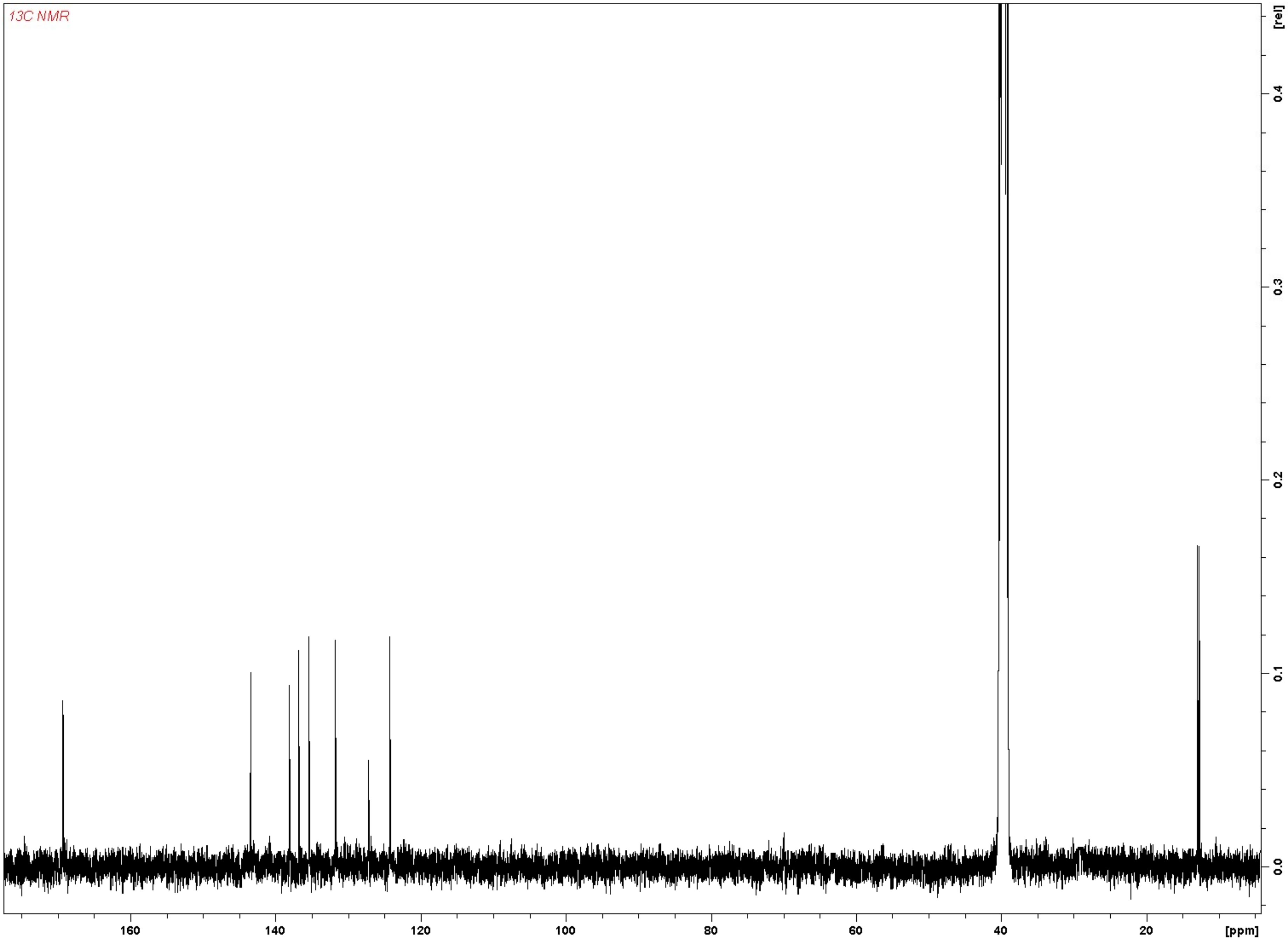

Figure S11. ^{1}H NMR spectrum of 13-*cis* crocetin (**6**) in CD_3OD.

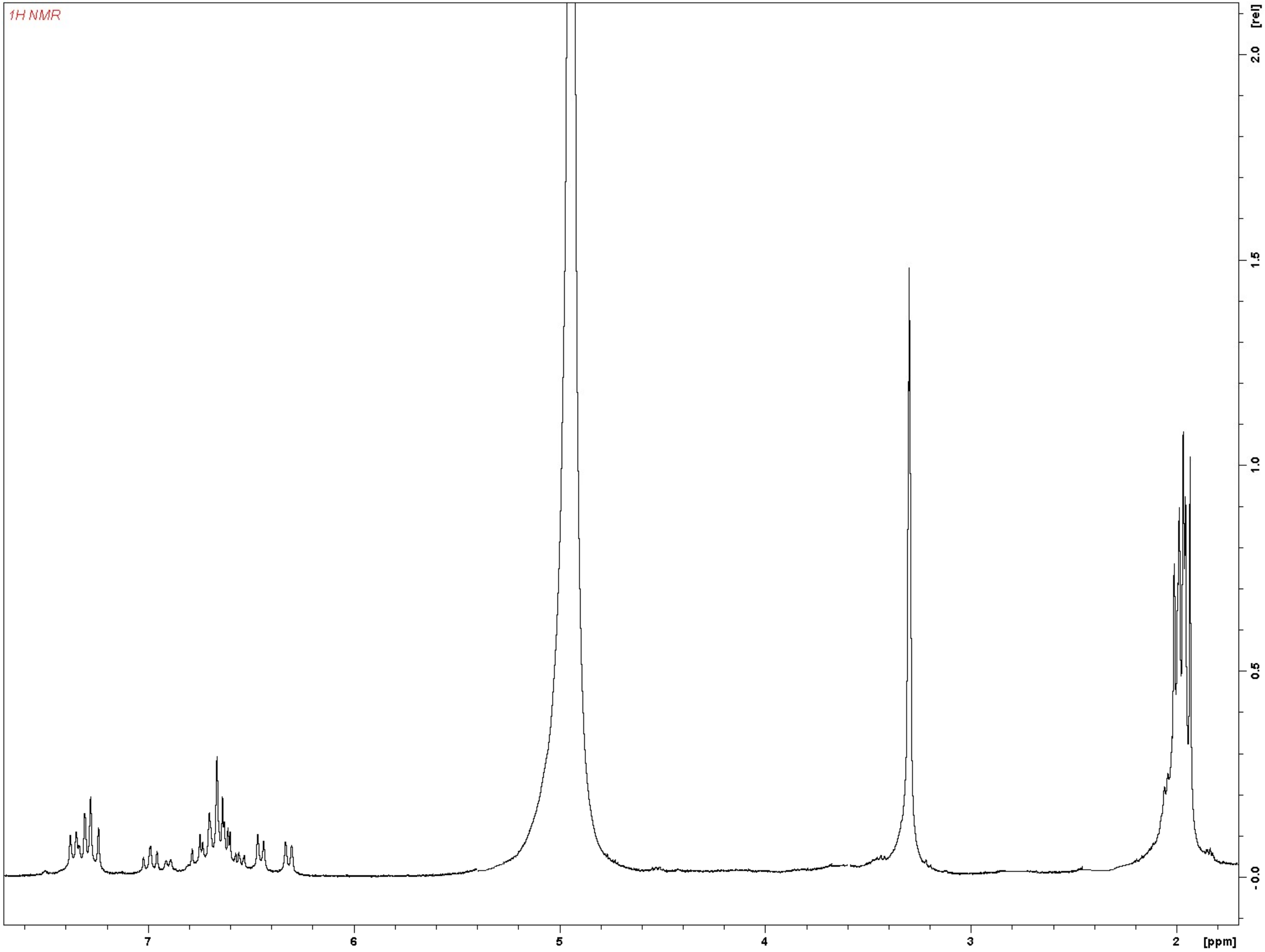

Figure S12. ^{13}C NMR spectrum of 13-*cis* crocetin (**6**) in CD_3OD.

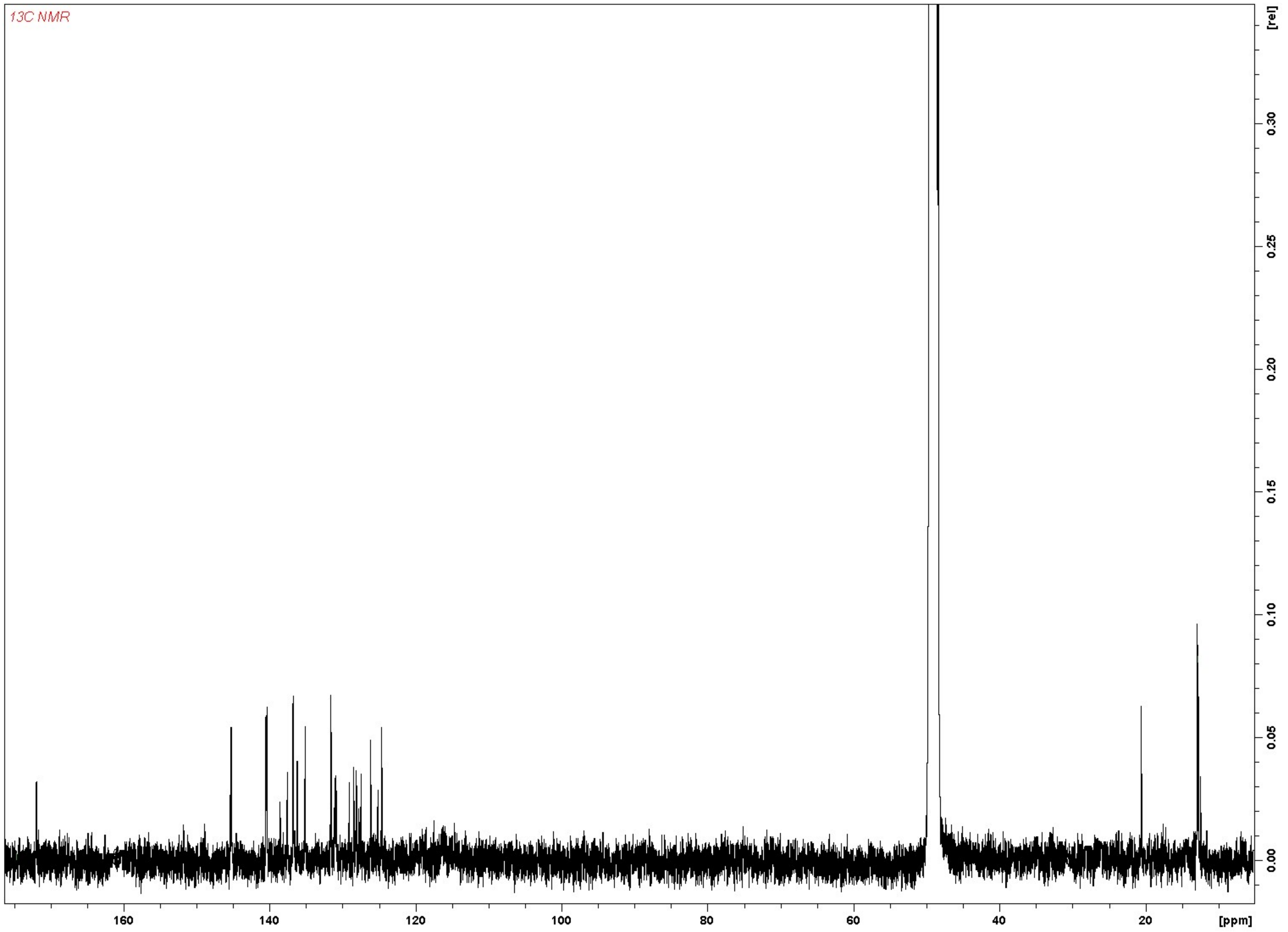

Figure S13. ^{1}H NMR spectrum of crocetin neapolitanosyl ester (**7**) in CD_3OD.

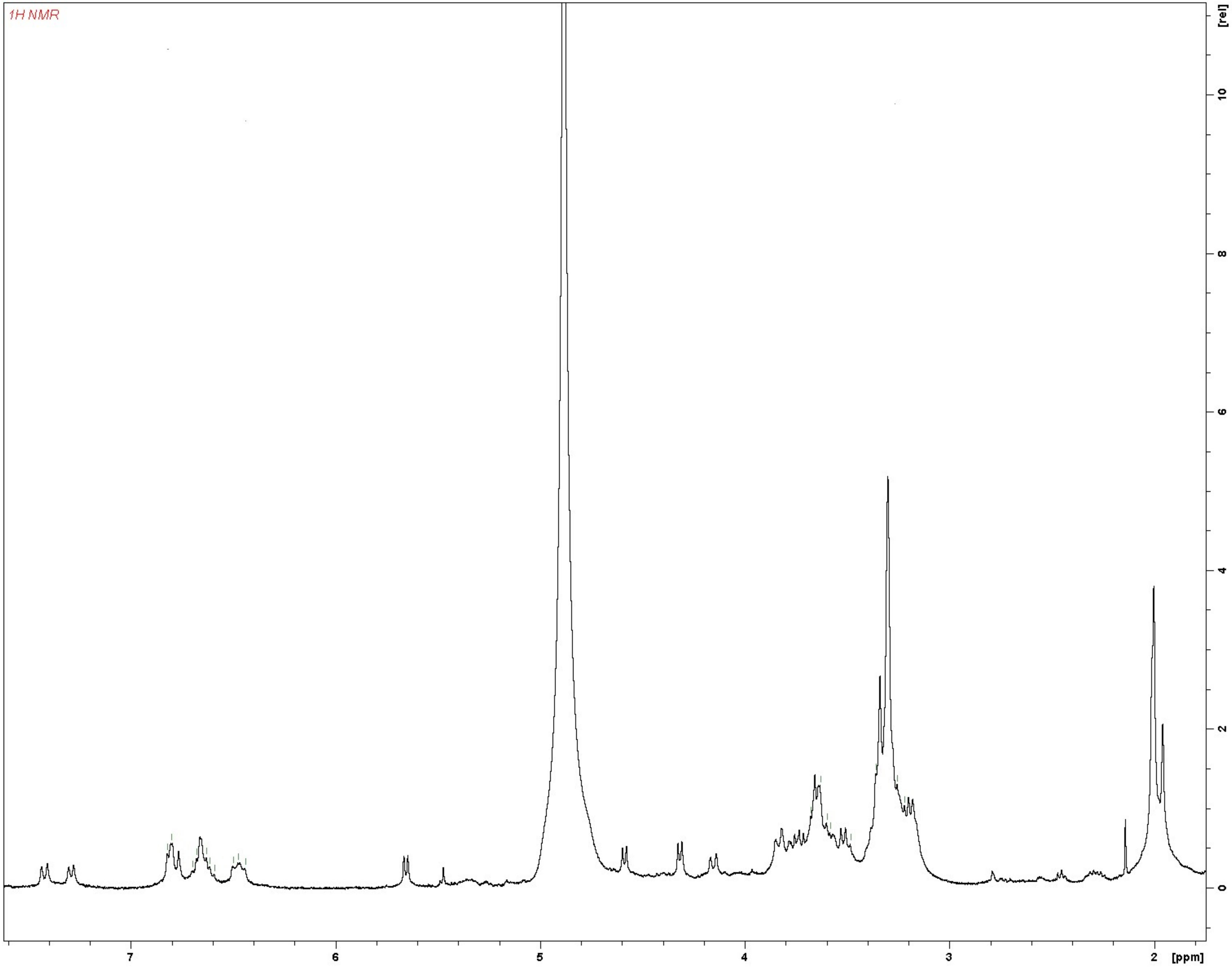

Figure S14. ^{13}C NMR spectrum of crocetin neapolitanosyl ester (**7**) in CD_3OD.

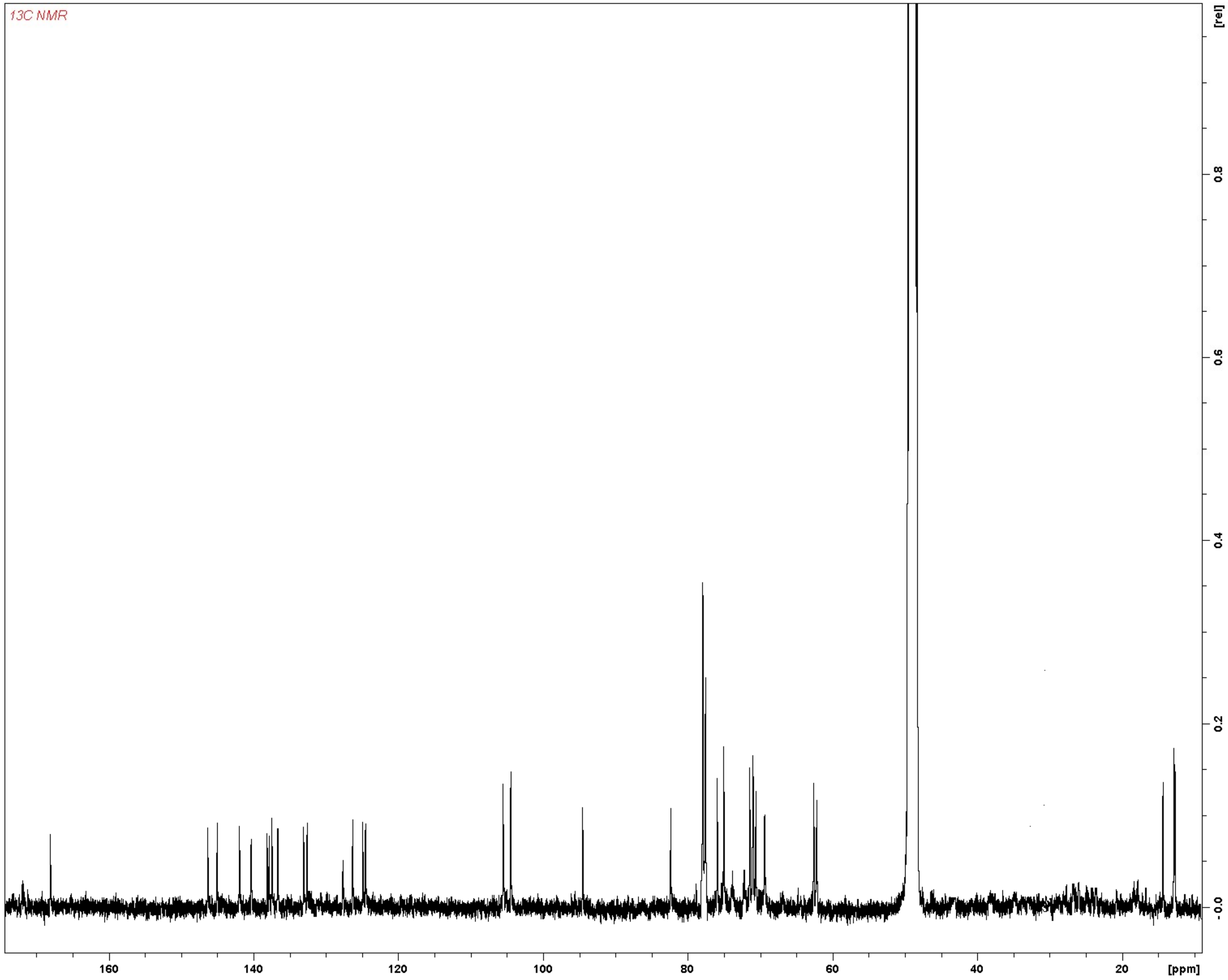

Figure S15. ^{1}H NMR spectrum of crocetin dineapolitanosyl ester (**8**) in CD_3OD-D_2O (1:1).

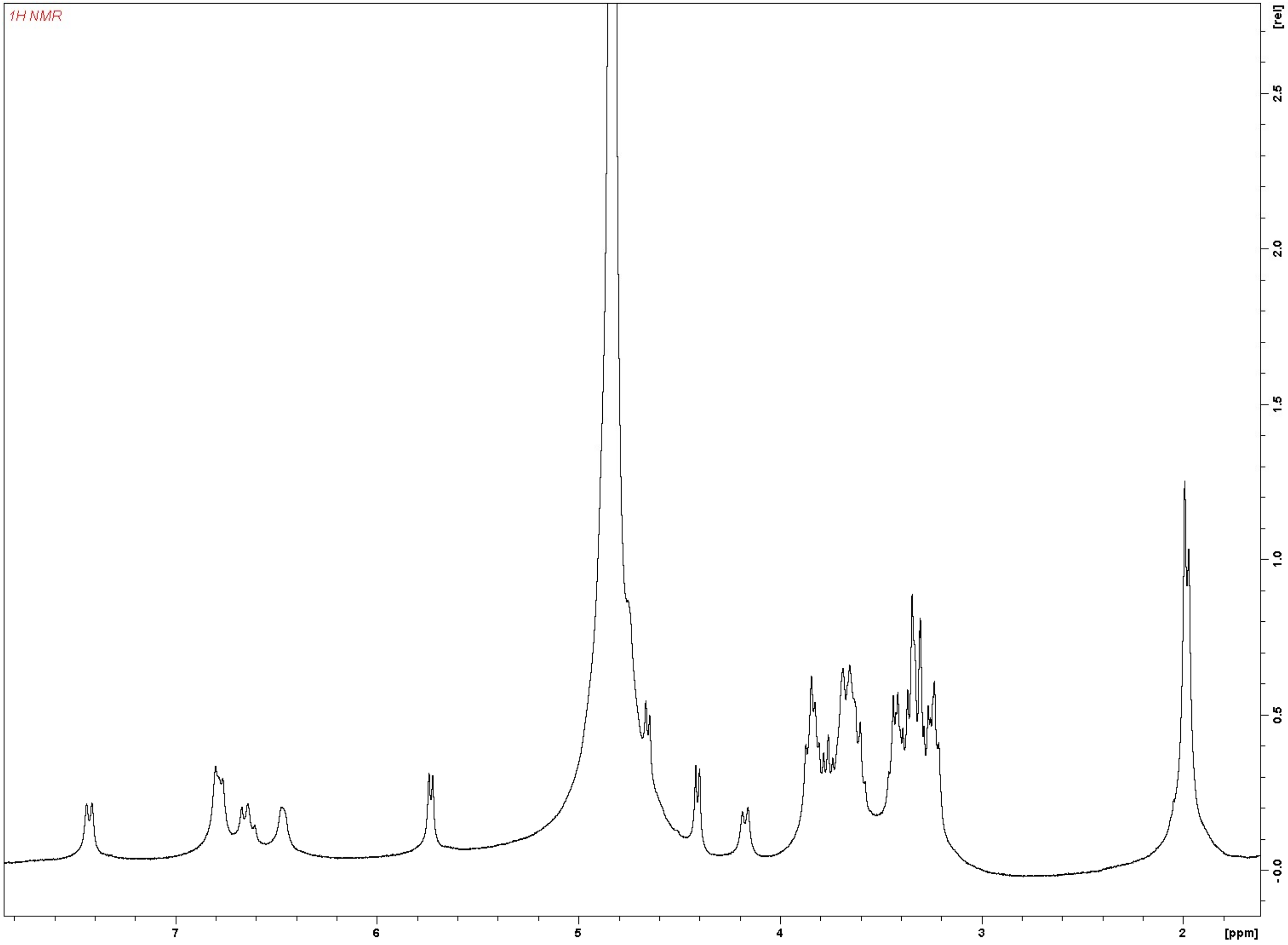

Figure S16. ^{13}C NMR spectrum of crocetin dineapolitanosyl ester (**8**) in CD_3OD.

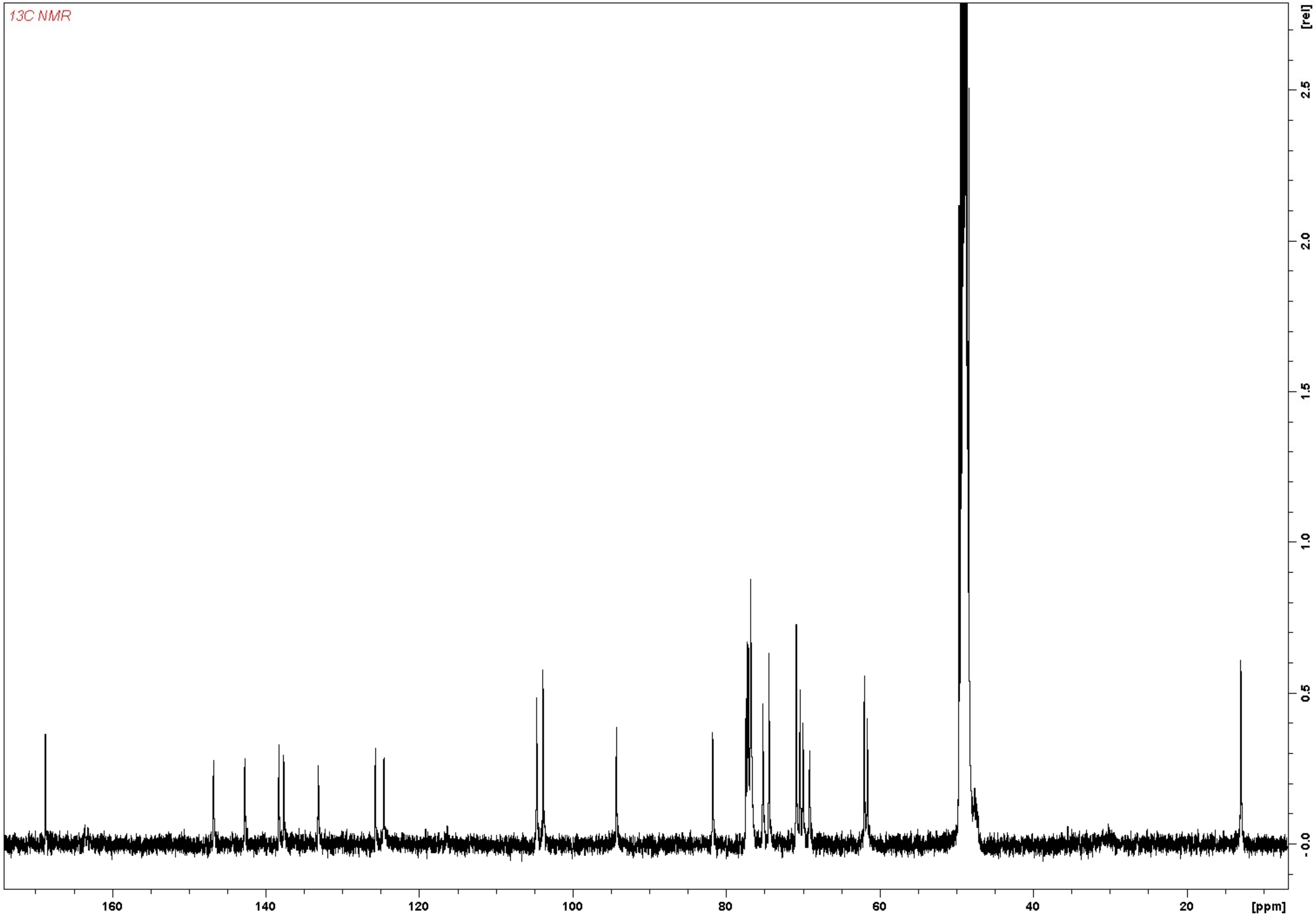

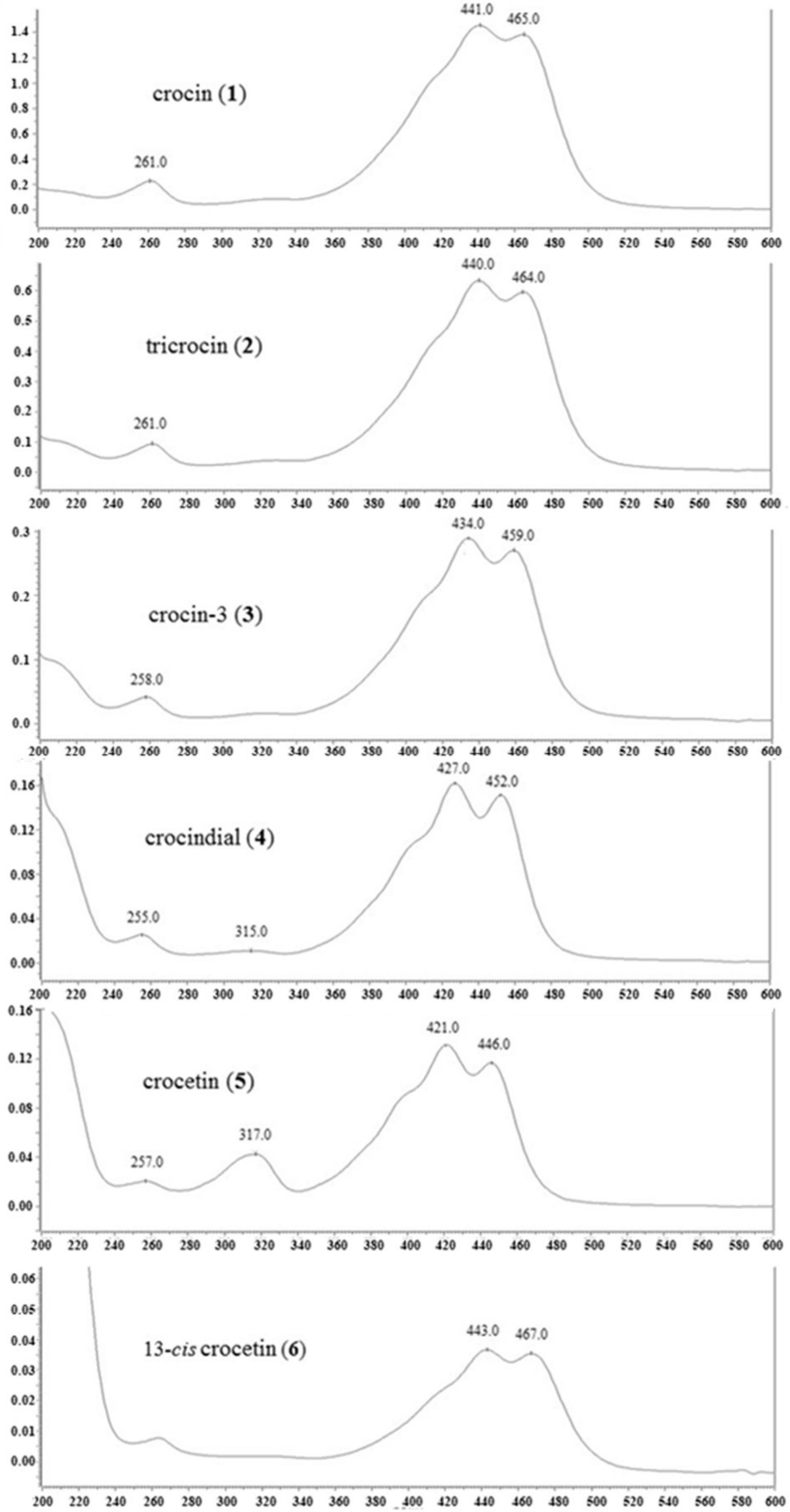

Figure S17. UV-Vis spectra of crocin (**1**), tricrocin (**2**), crocin-3 (**3**), crocetindial (**4**),crocetin (**5**), and 13-*cis* crocetin (**6**) in HPLC-DAD analysis.

Figure S18. Changes of crocin (**1**) and crocetin (**5**) through the grilled cooking model.

Experiment 1.
Crocin (**1**) (0.2 mg) in oil (5 mL) were heated at 180℃ for 5 min, and change of **1** was analyzed by HPLC.
(The conditions for HPLC were identical to those described in MATERIAL AND METHODS)

Experimental 1)

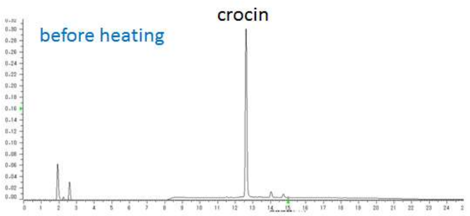

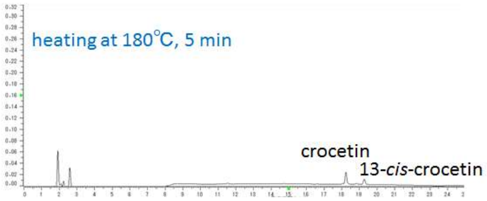

[Result]
The peak of **1** disappeared by heating, and the peaks of crocetin (**5**) and 13-*cis* crocetin (**6**) were observed.

Considering peak areas of **1**, **5**, and **6**, 95% of **1** was decomposed and 5% of **1** was changed into **5** and **6**. The peak area ratio of **5**:**6** was 10:1, approximately.

Experiment 2.
Crocetin (**5**) (0.1 mg) in oil (5 mL) were heated at 180℃ for 5 min, and change of **5** was analyzed by HPLC.
(The conditions for HPLC were identical to those described in MATERIAL AND METHODS)

Experiment 2)

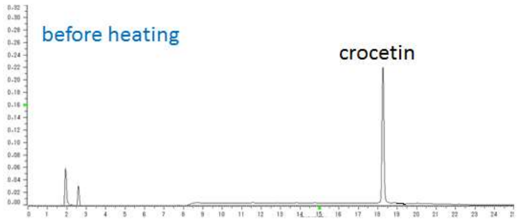

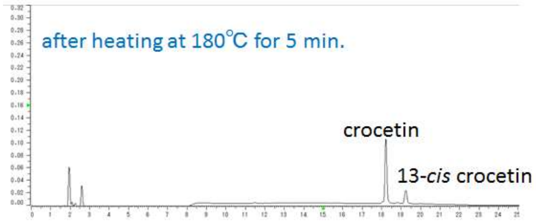

[Result]
The peak area of **5** decreased to 40%, and 13-*cis* crocetin (**6**) peak was observed by heating. The peak area ratio of **5**:**6** was 10:1, approximately.
